# Evaluation of the safety and functional effects of recombinant humanized type III collagen in food toxicology

**DOI:** 10.3389/fmed.2026.1765276

**Published:** 2026-02-12

**Authors:** Zhiyu Zhang, Wenqing Yang, Jingbo Yang, Xiaobin Lan, Chuanxiu Chen

**Affiliations:** Shanxi Jinbo Bio-Pharmaceutical Co., Ltd., Taiyuan, Shanxi, China

**Keywords:** 90-day toxicity study, anti-aging, anti-glycation, anti-inflammatory, antioxidant, genotoxicity, recombinant humanized type III collagen

## Abstract

This study aimed to comprehensively assess the edible safety and in vivo bioactivities of recombinant humanized type III collagen (rhCol III). A systematic toxicological evaluation was conducted, including subchronic toxicity tests, bacterial reverse mutation assays, mammalian erythrocyte micronucleus tests, and chromosome aberration tests. Concurrently, efficacy was evaluated using a zebrafish model to investigate multiple biological endpoints. The safety assessment revealed no toxicologically significant abnormalities, confirming a favorable safety profile for long-term oral consumption. In the zebrafish model, rhCol III significantly inhibited the expression of inflammatory factors, reduced the accumulation of advanced glycation end products (AGEs), alleviated oxidative stress damage, and improved molecular indicators associated with skin barrier function. The results demonstrate that rhCol III possesses multifaceted bioactivities, including anti-inflammatory, anti-glycation, antioxidant, and anti-aging properties. This research provides a robust scientific foundation for the development and application of rhCol III in high-value-added oral nutricosmetic products.

## 1 Introduction

Skin health and youthfulness act as a visible proxy for systemic physiological function. The process of skin aging is hallmarked not just by apparent changes (e.g., wrinkles, laxity) but also by underlying molecular-level mechanisms like oxidative stress, chronic inflammation, glycation reactions, and extracellular matrix degradation (1–4). Against this backdrop, the proliferation of the oral beauty market is underpinned by the prevailing paradigm of “internal care and external beauty.” Within this market, collagen has become a focal point of interest due to its pivotal role in preserving skin structural integrity and function (5–7).

Type III collagen (Col III), the second most prevalent structural protein (5–20% of total collagen) after type I, is abundant in elastic tissues including skin and blood vessels (1, 8) and is crucial for the proper assembly and mechanical stability of collagen fibrils. A deficiency in Col III results in structural abnormalities that undermine skin elasticity and healing (9, 10). Nevertheless, collagen sourced from animals presents significant limitations, such as risks of pathogen transmission, religious prohibitions, potential immunogenicity, and suboptimal biocompatibility (11, 12). Given these constraints, developing novel, safer collagen sources with improved compatibility for human physiology has emerged as a critical research frontier (13).

Significant progress has been made in producing recombinant humanized type III collagen (rhCol III) via genetic engineering (12, 14). Featuring an amino acid sequence highly similar to its natural human counterpart (15, 16), this protein exhibits excellent biocompatibility, low immunogenicity, and avoids risks inherent to animal-sourced collagen, showing great potential in pharmaceutical, cosmetic, and food industries (17–22). Given this intended use as a food ingredient for long-term oral consumption, a rigorous toxicological safety evaluation is essential before it can be approved (23). Consequently, a battery of tests — including genetic toxicity assessments (bacterial reverse mutation, mammalian chromosome aberration, and micronucleus tests) and a subchronic oral toxicity study — constitutes the essential scientific basis and a mandatory step toward its commercialization. While confirming safety is indispensable, scientifically validating its biological efficacy is equally critical. The zebrafish model, owing to its high genetic homology with humans, transparent embryos for easy observation, and short experimental cycle, has emerged as an ideal platform for evaluating the *in vivo* effects of functional food ingredients (24, 25).

This research systematically validated the edible safety and multi-faceted skin-protective efficacy of rhCol III, including anti-inflammatory, anti-glycation, antioxidant, and anti-aging activities, through a complete toxicological evaluation system and a zebrafish efficacy model, thereby providing scientific evidence for the development of high-end oral nutricosmetic products.

## 2 Materials and methods

The recombinant humanized type III collagen (rhCol III) used in this study was provided by Shanxi Jinbo Bio-Pharmaceutical Co., Ltd., with a purity of ≥ 80% and a molecular weight of approximately 45 kDa.

The Specific Pathogen Free Sprague–Dawley (SPF SD) rats used in the 90-day oral toxicity test were from Hunan Slake Jingda Laboratory Animal Co., Ltd. (ethical approval number no.: JC20220100-M4), and all the animals underwent a 3-day quarantine and adaptive feeding.

The SPF mouse used in the genotoxicity test were supplied by Changsha Tianqin Biotechnology Co., Ltd. (ethical approval number no. JC20220100-G2), and quarantined and acclimated for 3 days.

Sample sizes for the 90-day toxicity study and genotoxicity assays were selected accordingto relevant OECD test guidelines; zebrafish group sizes were chosen based on common practice and pilot variability, and experiments were independently replicated to ensure robustness. Relevant data are provided in Appendix C.

### 2.1 General toxicity

#### 2.1.1 Subchronic toxicity study (90-day feeding test)

Instruments: electronic analytical balance (JA3003), electronic balance (TP-3KE), biological microscope (Olympus), automated biochemical analyzer (BS-600), veterinary automated blood cell analyzer (BC-5300 Vet), automated coagulation analyzer (RAC-1830), automated electrolyte analyzer (XI-1021), urine 11-parameter analyzer (Enokobo 500), water purification system (ELGA Pure Chorus1 Complete), automatic staining machine (Tissue-Tek Prisma^®^ Plus), automatic dehydration machine (HP300/HP300 Plus), electronic rotary microtome (HM-340E) and other pathological examination instruments, etc.

Reagents: Biochemical kit (Shenzhen Mindray Biomedical Electronics Co., Ltd.), K/Na/Cl reagent package (Medica Medical Equipment (Suzhou) Co., Ltd.), Hematology kit (Shenzhen Mindray Biomedical Electronics Co., Ltd.), and Chloral hydrate (Tianjin Keminoh Chemical Reagent Co., Ltd.).

#### 2.1.2 Dose determination

The median lethal dose (LD_50_) of the sample is higher than 10,000 mg/kg bw, therefore the sample is considered practically non-toxic. The expected recommended daily intake for the human body is 70 mg (calculated based on a 70-kg adult, 1 mg/kg bw/d). According to the Chinese national standard, the highest experimental dose was set at 100-fold the expected recommended intake dose, 100 mg/kg bw/day.

#### 2.1.3 Animals

The SPF SD rats with half male and half female were selected for the experiment, and the female rats nulliparous and non-pregnant. At the beginning of the experiment, the range of weight was between 70.0–91.0 g (female between 70.0–89.5 g and male between 70.3–91.0 g). Each experiment rat was housed individually in cages with ad libitum access to standard diet and water.

#### 2.1.4 Research design

Three dose-level groups and one control group were established based on the Chinese national standard. Each group has 20 rats (10/sex). Based on the maximum experimental dose, the medium group and the low dose group were designed at twice the intervals (high-dose groups, 100 mg/kg bw; intermediate-dose groups, 50 mg/kg bw; low-dose groups, 25 mg/kg bw and control groups, 0 mg/kg bw). In addition, the study established an interim control satellite group, an interim high-dose satellite group, a recovery control satellite group, and a recovery high-dose satellite group to facilitate observation during the mid-experimental and recovery periods (10 rats/group; 5/sex).

The freshly prepared collagen aqueous solutions were administered to the rats at 10 mL/kg bw by oral gavage. According to the designed dosages, 0.05 g, 0.10 g, and 0.40 g samples were weighed and adjusted to 20 mL, 20 mL, and 40 mL for use by the low-dose, intermediate-dose, and high-dose animal groups, respectively. The corresponding volume of the test solution was prepared every day based on the weight of the rats. Dose formulations were administered once daily for 90 days (6 days /week). The control group and its satellite group were given the same volume of purified water.

##### 2.1.4.1 Clinical signs

The general signs, behaviors, poisoning symptoms and mortality of all rats were observed daily.

##### 2.1.4.2 Body weight and food consumption

The body weight and the food intake of rats were measured once per week (twice weekly during the first 4 weeks), and the weekly food utilization rate, total food utilization rate, total food intake, and total weight gain were calculated.

##### 2.1.4.3 Ophthalmological examination

Ophthalmoscopical examinations of the cornea, lens, bulbar conjunctiva, and iris were conducted on rats from the high-dose group, control group, recovery high-dose satellite group, and recovery control satellite group prior to the initiation of the experiment and upon its completion.

##### 2.1.4.4 Hematological indicators and urinalysis indicators

Blood samples were collected from the abdominal aorta under anesthesia after approximately 16 h of fasting at the interim time point (satellite groups), after the experimental period and after the recovery periods (satellite groups), and analyzed for the following parameters: red blood cell (RBC), hematocrit (HCT), hemoglobin (HGB), platelets (PLT), prothrombin time (PT), activated partial thromboplastin time (APTT), white blood cell (WBC), and other items.

Some blood biochemical indicators were also investigated: alanine transaminase (ALT), aspartate transaminase (AST), alkaline phosphatase (ALP), gamma-glutamyl transferase (GGT), total protein (TP), albumin (Alb), total cholesterol (TC), triglycerides (TG), urea, creatinine (Cr), glucose (GLU), potassium (K^+^), sodium ion (Na^+^), chloride (Cl^−^), and other items.

The urine was collected at the same time period as the blood and analyzed for the following urinalysis indicators: pH, protein (PRO), glucose (GLU), specific gravity (SG), ketone (KET, acetoacetic acid).

##### 2.1.4.5 Necropsy and organ weight

Animals underwent necropsy after the experiment (after the recovery for satellite groups), including skin, skull, thorax, abdomen and internal organs.

Organs, including the brain, heart, thymus, adrenal gland, liver, kidney, spleen, testis, epididymis, uterus, and ovary, were weighed, and the relative weights (organ/body ratio) were calculated.

##### 2.1.4.6 Histopathology

All the tissues and organs of the animals, including the brain, pituitary gland, thyroid gland, thymus, lung, heart, liver, spleen, kidney, adrenal gland, stomach, duodenum, jejunum, ileum, colon, rectum, ovary, uterus, testis, epididymis, prostate, bladder, pancreas, and lymph nodes, were fixed with a 10% formaldehyde solution and subjected to histopathological examination.

##### 2.1.4.7 Statistical analysis

The data were statistically analyzed using IBM SPSS Statistics 20.0 software.

For the control group and dose groups, continuous data (body weight, food consumption, food utilization rate, hematology, serum biochemistry, coagulation function, electrolytes, organ weights, and organ-to-body weight ratios) were analyzed by one-way ANOVA. Homogeneity of variance was assessed using a variance homogeneity test. When variances were homogeneous (*p* > 0.05) and the overall difference was significant (*p* < 0.05), Dunnett’s test was applied for multiple comparisons versus the control; when variances were heterogeneous (*p* < 0.05), the Games–Howell test was used for multiple comparisons. Urinalysis indices were analyzed using a rank-sum test.

For the control satellite group and high-dose satellite group, the same continuous variables were compared using an independent-samples t-test, with interpretation based on variance homogeneity (*p* > 0.05) or heterogeneity (*p* < 0.05). Urinalysis indices in satellite groups were analyzed using a rank-sum test or a chi-square test. Abnormal findings from gross necropsy and histopathology were analyzed using a chi-square test, and *p* < 0.05 was considered statistically significant.

### 2.2 Genotoxicity studies

#### 2.2.1 Bacterial reverse mutation assay (Ames test)

##### 2.2.1.1 Instruments

Electronic analytical balance (JA3003), Olympus biological microscope (CX43), and biological safety cabinet (Thermo Fisher 1,379).

##### 2.2.1.2 Reagents

Rat liver microsomal enzyme S9 (Wuxi Xinrun Biotechnology Co., Ltd., batch no.: 20221108), glucose-6-phosphate sodium salt (BOMEI, batch no.: Y004669), oxidized coenzyme II (BOMEI, batch no.: G7250), TA97a, TA98, TA100, TA102, TA1535 (MOLTOX, batch no.: 5574D, 5602D, 5594D, 5585D, 5573D), DMSO (MP Biomedicals, LLC, batch no.: YC0403), fenaminosulf (Sigma, batch no.: BCBW1260), Sodium azide (ACROS, batch no.: A0345092), 2-Aminofluorene (Fluka, batch no.: 252361689), and 1,8-Dihydroxyanthraquinone (Shanghai Jingchun Reagent Co., Ltd., batch no.: 200808054).

A pilot test was first conducted. Using purified water as the solvent, four dose groups of 5,000, 2000, 1,000, and 500 μg/plate were tested under the −S9 condition. The results showed no precipitation of the test substance in any dose group, and the background bacterial lawn growth showed no significant difference compared to the control group. This indicated that the test substance had no bacterial toxicity at concentrations up to 5,000 μg/plate.

Based on pilot test, the formal test established five dose groups of 5,000, 1,582, 500, 158, and 50 μg/plate, along with an untreated control group, a solvent control group (purified water and DMSO), and a positive mutagen control group. During the preparation of the test substance, 0.5 g of the sample was accurately weighed and diluted to 10 mL with purified water to obtain the highest dose suspension of 50 mg/mL. The prepared test solution was sterilized at 0.103 MPa for 20 min for later use. Other dose group solutions were then prepared through serial dilution, with concentrations of 15.82, 5, 1.58, and 0.5 mg/mL, respectively. The verification test included five dose groups of 5,000, 1,000, 200, 40, and 8 μg/plate, with all other experimental conditions remaining unchanged.

During the formal experiment, the cryopreserved bacterial strain culture was inoculated into nutrient broth medium and cultured at 37 °C with shaking at 100 oscillations per minute for 10 h, achieving a viable count of ≥ 1 × 10^9^ CFU/mL. The test was performed under both −S9 and +S9 conditions: 0.1 mL of the bacterial inoculum, 0.1 mL of the test substance solution, and, for metabolic activation (+S9), an additional 0.5 mL of 10% S9 mix were added to 2 mL of top agar medium that had been melted and maintained at 45 °C in a water bath. The mixture was thoroughly mixed, quickly poured onto the base agar plate, and evenly spread. Each dose was tested in triplicate under both metabolic activation conditions. A positive control group (with the same volume of standard mutagen), a solvent control group (with the same volume of solvent only), and an untreated control group (with bacterial inoculum only) were also included, with all other procedures consistent with the experimental group. After incubation at 37 °C for 48 h, the number of revertant colonies per plate was directly counted. The mean and standard deviation of the number of revertant colonies from the triplicate plates in each group were calculated using Microsoft Excel 2007 software.

#### 2.2.2 The mammalian *in vivo* micronucleus test

Instruments: Electronic balance (TP-3KE), electronic analytical balance (JA3003), pure water machine (ELGA Pure Chorus1 Complete), and Olympus biological microscope (CX43).

Reagents: Giemsa stock solution (3.8 g of Giemsa stain, 375 mL of methanol, and 125 mL of glycerol. Giemsa stain was ground in a mortar with a small amount of methanol until very fine and diluted with methanol to 375 mL. Once the stain was completely dissolved, 125 mL of glycerol was added, and the solution was mixed well. The solution was stored in a 37 °C incubator for 48 h, during which it was shaken several times to ensure complete dissolution of the stain. After removal and filtration, the solution was stored for long-term use), inactivated fetal bovine serum (fetal bovine serum was filter sterilized and inactivated in a 56 °C water bath for 30 min. Inactivated serum was usually stored in a 4 °C refrigerator), and cyclophosphamide for injection (Baxter).

Based on the acute oral LD_50_ (> 10,000 mg/kg bw) of rhCol III determined in our laboratory in mice three dose groups were designed as follows: high dose (10,000 mg/kg bw), intermediate dose (5,000 mg/kg bw), and low dose (2,500 mg/kg bw), along with a vehicle control group (pure water) and a positive control group (cyclophosphamide at 40 mg/kg bw). The test sample solutions were prepared by weighing 1.25 g, 2.5 g and 5.0 g of rhCol III, respectively, adding pure water to a final volume of 10 mL (resulting in concentrations of 125 mg/mL, 250 mg/mL and 500 mg/mL, corresponding to the 2,500 mg/kg, 5,000 mg/kg and 10,000 mg/kg body weight dose groups). The positive control solution was prepared by dissolving 20 mg of cyclophosphamide in 10 mL of sodium chloride for injection, yielding a concentration of 2.0 mg/mL.

Animals received oral gavage administration at a volume of 20 mL/kg bw using a “30-h dosing regimen”: two doses were administered with a 24-h interval, and bone marrow was collected 6 h after the second gavage. During the experiment, animals were observed daily for general conditions, including skin, hair, eyes, mucous membranes, respiratory function, circulatory status, neurological activity, limb movements, and behavioral abnormalities.

Six hours after the final gavage, animals were euthanized via cervical dislocation. The sternum was excised, surface blood and muscle were removed, and the bone marrow cavity was exposed by transverse sectioning. Bone marrow fluid was expressed from the cavity using a small hemostat, dropped onto a coverslip pre-coated with fetal bovine serum, and mixed thoroughly. A smear was prepared by dragging a slide at a 45° angle over the coverslip toward the tail, forming a tongue-shaped film. The smear was air-dried, fixed with methanol for 5–10 min, and stained with a freshly prepared 10% Giemsa working solution (prepared by mixing Giemsa stock solution with 1/15 mol/L PBS at a 1:9 volume ratio, pH 6.8) for 10–15 min. After rinsing with purified water, the smear was air-dried again.

For microscopic examination, low-power lenses were used to select areas with uniformly distributed and well-stained cells. Under oil immersion, polychromatic erythrocytes (PCEs, gray-blue) and normochromatic erythrocytes (NCEs, pale orange-red) were observed. For each animal, 2000 PCEs were counted to determine the number of PCEs containing micronuclei (micronuclei: single, round, with regular edges, staining consistency with nuclear material, and a diameter 1/20–1/5 that of erythrocytes). The micronucleus frequency was calculated as (number of micronucleated PCEs/total number of PCEs) × 1,000‰. Additionally, 200 total erythrocytes (PCEs + NCEs) were counted to calculate the ratio of immature erythrocytes, expressed as (number of PCEs/total number of erythrocytes) × 100%.

Statistical Analysis: Data were statistically analyzed using IBM SPSS Statistics 20.0. Animal body weight, frequency of micronuclei, and the proportion of immature erythrocytes in total erythrocytes were compared between each dose group and the vehicle control group using t-tests and/or one-way ANOVA, and *p* < 0.05 was considered statistically significant.

#### 2.2.3 *In vitro* mammalian cell chromosome aberration assay

Instruments: Electronic analytical balance (JA3003), Olympus biological microscope (CX43), and ultraclean bench (YJ-1300).

Reagents: DMEM high glucose (Zhejiang GinomBiomedical Technology Co., Ltd., batch no.: 22112501), fetal bovine serum (Zhengzhou Yikan Biological Engineering Co., Ltd., batch no.: 202105026), penicillin–streptomycin solution (Zhejiang GinomBiomedical Technology Co., Ltd., batch no.: 22062302), trypsin–EDTA solution (Zhejiang GinomBiomedical Technology Co., Ltd., batch no.: 22071201), rat liver microsomal enzyme S9 (Wuxi Xinrun Biotechnology Co., Ltd., batch no.: 20221108), glucose-6-phosphate sodium salt (BOMEI, batch no.: Y004669), and oxidized coenzyme II (BOMEI, batch no.: G7250).

Dose design: A pilot test was conducted using DMEM as the vehicle. CHL cells were exposed to the test article at 500, 1,000, 2,000, and 5,000 μg/mL under the −S9 condition for 4 h; no precipitation was observed, and cell viability was 97.65, 99.06, 102.82, and 94.37%, respectively, at the end of the culture period. A second pilot exposure was performed under the −S9 condition for 24 h at the same dose levels; no precipitation was observed, and cell viability was 100.40, 98.39, 99.19, and 100%, respectively. Based on these results, three dose levels (1,250, 2,500, and 5,000 μg/mL) were selected for the formal test, together with a vehicle control (DMEM) and positive controls (20 μg/mL MMS for −S9; 10 μg/mL CP for +S9).

The cell line selection and study design were in accordance with Chinese national standards. Chinese hamster lung (CHL) cells were used, and a rat liver S9 mix (10% S9), induced by sodium phenobarbital and *β*-naphthoflavone, served as the *in vitro* metabolic activation system. Prior to the formal assay, a preliminary range-finding test was conducted using DMEM as the vehicle. Based on solubility and cell viability, three concentrations (1,250, 2,500, and 5,000 μg/mL) were selected for the definitive assay. A vehicle control and positive controls were included. Each group was evaluated under both −S9 and +S9 conditions. Cyclophosphamide (CP, 10 μg/mL) and methyl methanesulfonate (MMS, 20 μg/mL) were used as positive controls for +S9 and −S9 conditions, respectively.

Adjust the density of the cell suspension to 1 × 10^6^ cells/mL. Add 2 mL of the cell suspension and 3 mL of complete culture medium (containing 10% fetal bovine serum and 100 IU/mL penicillin–streptomycin) to each culture flask, ensuring the number of cells in each flask reaches 2 × 10^6^. Place the flasks in an incubator at 37 °C with 5% CO_2_ for 24 h. After incubation, aspirate the culture medium from the flasks, then add 50 μL of the test solution (add DMEM for the solvent control group and the corresponding positive control solution for the positive control group), 4.45 mL of serum-free culture medium, and 0.5 mL of S9 mixture (if S9 mixture is not added, make up the volume with serum-free culture medium). Incubate the flasks at 37 °C for 4 h. After 4 h, aspirate the culture medium containing the test substance, replace it with 5 mL of complete culture medium, and continue incubating at 37 °C until 24 h (from the start of the second incubation phase).

Cell division was blocked at metaphase by adding 50 μL of 100 μg/mL colchicine (final concentration 1 μg/mL) to the culture flask 4 h before the end of the culture. After the colchicine has acted for 4 h, digest the cells with a 0.25% trypsin solution. Subsequently, centrifuge to harvest the cells and prepare cell slides. Observe the spreads under an oil immersion lens and well-dispersed metaphase chromosome spreads were selected for analysis (100 spreads per group), and record the types of chromosomal aberrations and their quantities (when two or more aberrations appear in one cell, the number of abnormal cells is counted as 1).

Statistical Analysis: Chromosome aberration frequencies were statistically analyzed across groups using the χ^2^ test with IBM SPSS Statistics 20.0 software, at a significance level of *α* = 0.05.

Verification Experiment: Under the condition of no S9 mixture added, extend the exposure time of the test substance to 24 h. Use 10 μg/mL MMS as the positive control, and repeat the experiment.

A positive result for the test substance in this experimental system was determined if either of the following two conditions was met:

- The test substance induced a statistically significant increase in the number of structural chromosomal aberrations, which was dose-dependent;
- The test substance elicited a statistically significant increase in the number of structural chromosomal aberrations at any single dose, and this effect was reproducible.

### 2.3 Potential oral beauty efficacy

#### 2.3.1 Anti-aging action

##### 2.3.1.1 Test Animals

AB strain wild-type zebrafish. The ambient temperature of the laboratory was maintained at 28 °C ± 1 °C, with relative humidity below 75%. A programmable lighting control system and a dedicated fresh air system were installed. The temperature of the rearing water was precisely regulated at 28 °C ± 0.5 °C.

##### 2.3.1.2 Instruments

Electronic analytical balance (JA3003), stereoscopic fluorescence microscope (Leica MZ10 F).

##### 2.3.1.3 Reagents

Modeling agent (20 mg/mL D-galactose, dissolved in standard laboratory pure water), positive control sample (4 μmol/L vitamin E, dissolved in DMSO), experimental collagen samples (three gradient concentrations of collagen prepared with standard laboratory pure water as solvent: 5 μg/mL, 50 μg/mL, 500 μg/mL). The experimental dosage design preliminary experiment was conducted based on *OECD. 2013. Test No. 236: Fish Embryo Acute Toxicity (FET) Test.*

The experiment was divided into four groups: blank control group, modeling group (modeling agent), experimental group (modeling agent and sample), and positive control group (modeling agent and positive control sample). The blank control group received no treatment; the model control group was induced to establish an aging model using D-galactose; and the experimental group was treated with the test sample after D-galactose induction.

Normally developed 6-h-post-fertilization (6 hpf) zebrafish larvae were selected and randomly assigned to 6-well cell culture plates, with 30 embryos per well. The standard dilution water in the 6-well plate was removed without harming the embryos, and 3 mL of the test substance dilution at the corresponding concentration was quickly added to each well. Each group was set up with 3 replicates. After thorough mixing, the culture plate lid was closed, and the plate was wrapped with aluminum foil. Incubation was carried out in a biochemical incubator at 28.5 °C ± 1.0 °C in the dark until the endpoint (5 dpf).

After incubation, zebrafish were stained using a *β*-galactosidase staining kit. At least 10 zebrafish with normal phenotypes and behaviors were randomly selected, and the intensity of blue staining was analyzed.

The statistical results of the experiment were expressed as mean ± standard deviation (SD), and *p* < 0.05 was used as the criterion for determining that the difference was statistically significant.

#### 2.3.2 Anti-glycation action

In this experiment, the formation of advanced glycation end products (AGEs) in zebrafish was induced in a high-glucose environment. Subsequently, zebrafish were administered rhCol III, and its anti-glycation effect was observed. Since AGEs possess fluorescent properties, the final quantification of *in vivo* AGEs was evaluated via fluorescence detection.

##### 2.3.2.1 Test Animals

AB strain wild-type zebrafish. The ambient temperature of the laboratory was maintained at 28 °C ± 1 °C, with relative humidity below 75%. A programmable lighting control system and a dedicated fresh air system were installed. The temperature of the rearing water was precisely regulated at 28 °C ± 0.5 °C.

##### 2.3.2.2 Instruments

Electronic analytical balance (JA3003), stereoscopic fluorescence microscope (Leica MZ10 F), microplate reader (Varioskan LUX).

##### 2.3.2.3 Reagents

Modeling agent (0.4 mol/L glucose, dissolved in standard laboratory pure water), positive control sample (20 mg/mL carnosine, dissolved in standard laboratory pure water), experimental collagen samples (three gradient concentrations of collagen prepared with standard laboratory pure water as the solvent: 5 μg/mL, 50 μg/mL, 250 μg/mL). The experimental dosage design preliminary experiment was conducted based on *OECD. 2013. Test No. 236: Fish Embryo Acute Toxicity (FET) Test*.

The experiment was divided into four groups: a blank control group, a modeling group (treated only with the modeling agent), an experimental group (treated with the modeling agent and experimental collagen sample), and a positive control group (treated with the modeling agent and positive control sample). Among them, the blank control group received no treatment; the model control group was induced to undergo glycation reaction using high-concentration glucose; and the experimental group and positive control group were administered the corresponding samples after treatment with high-concentration glucose.

In the experimental operation, normally developed 5-day-post-fertilization (5 dpf) zebrafish larvae were selected and randomly assigned to 6-well cell culture plates, with 30 larvae placed in each well. The standard dilution water in the plates was removed without damaging the larvae, and then 3 mL of the test substance dilution at the corresponding concentration was quickly added to each well. After thorough mixing, the culture plate lids were closed, the plates were wrapped with aluminum foil, and incubated in a biochemical incubator at 28.5 °C ± 1.0 °C in the dark.

During the incubation period, fresh test solution was replaced daily, and the incubation lasted for 3 consecutive days. After that, the zebrafish were treated in a shaker at 60 °C for 24 h, and the supernatant was collected by centrifugation.

The fluorescence intensity of the supernatant at 453 nm after excitation with 360 nm excitation light was measured, and this fluorescence intensity index was used to evaluate the anti-glycation efficacy.

The statistical results of the experiment were expressed as mean ± standard deviation (SD), and *p* < 0.05 was used as the criterion for determining that the difference was statistically significant.

#### 2.3.3 Anti-oxidation action

##### 2.3.3.1 Test Animals

AB strain wild-type zebrafish. The ambient temperature of the laboratory was maintained at 28 °C ± 1 °C, with relative humidity below 75%. A programmable lighting control system and a dedicated fresh air system were installed. The temperature of the rearing water was precisely regulated at 28 °C ± 0.5 °C.

##### 2.3.3.2 Instruments

Electronic analytical balance (JA3003), stereoscopic fluorescence microscope (Leica MZ10 F), microplate reader (Varioskan LUX).

##### 2.3.3.3 Reagents

Modeling agent (600 μmol/L hydrogen peroxide, solvent: standard laboratory pure water), positive control sample (3 μg/mL glutathione, solvent: standard laboratory pure water), experimental collagen samples (three gradient concentrations prepared with standard laboratory pure water as solvent: 5 μg/mL, 50 μg/mL, 250 μg/mL). The experimental dosage design preliminary experiment was conducted based on *OECD. 2013. Test No. 236: Fish Embryo Acute Toxicity (FET) Test*.

The experiment was divided into four groups: blank control group, model group (modeling agent), experimental group (modeling agent and sample), and positive control group (modeling agent and positive control sample). The blank control group received no treatment; the model control group was treated with hydrogen peroxide; the experimental group and positive control group were treated with hydrogen peroxide followed by the corresponding samples.

Normally developed 3-day-post-fertilization (3 dpf) zebrafish larvae were selected and randomly assigned to 6-well cell culture plates (30 larvae per well). The water in the 6-well plate was removed without harming the larvae, and 3 mL of the test substance dilution at the corresponding concentration was immediately added to each well. After thorough mixing, the plate was covered and wrapped with aluminum foil, then incubated in a biochemical incubator at (28.5 °C ± 1.0 °C) in the dark for 24 h.

After incubation, the larvae were transferred to a 96-well plate, and the permeable intracellular reactive oxygen species (ROS) indicator 2′,7’-Dichlorodihydrofluorescein diacetate (DCFH-DA) was added. The fluorescence intensity at 525 nm was measured after excitation with 488 nm excitation light, and this index was used to evaluate the antioxidant efficacy.

Statistical results are expressed as mean ± SD. *p* < 0.05 indicates a statistically significant difference.

#### 2.3.4 Anti-inflammatory action

##### 2.3.4.1 Test animals

Transgenic zebrafish with green fluorescent neutrophils. The ambient temperature of the laboratory was maintained at 28 °C ± 1 °C, with relative humidity below 75%. A programmable lighting control system and a dedicated fresh air system were installed. The temperature of the rearing water was precisely regulated at 28 °C ± 0.5 °C.

##### 2.3.4.2 Instruments

Electronic analytical balance (JA3003), stereoscopic fluorescence microscope (Leica MZ10 F).

##### 2.3.4.3 Reagents

Modeling agent (60 μg/mL sodium dodecyl sulfate, solvent: standard laboratory pure water), positive control sample (30 μg/mL indomethacin, dissolved according to the supplier’s instructions), experimental collagen samples (three gradient concentrations prepared with standard laboratory pure water as solvent: 10 μg/mL, 100 μg/mL, 500 μg/mL). The experimental dosage design preliminary experiment was conducted based on *OECD. 2013. Test No. 236: Fish Embryo Acute Toxicity (FET) Test*.

The experiment was divided into four groups: blank control group, model group (modeling agent), experimental group (modeling agent and sample), and positive control group (modeling agent and positive control sample). The blank control group received no treatment; the model control group was treated with SDS; the experimental group and positive control group were treated with SDS followed by the corresponding samples.

Normally developed 2-day-post-fertilization (2 dpf) transgenic zebrafish larvae with green fluorescent neutrophils were selected and randomly assigned to 6-well cell culture plates (30 larvae per well). The standard dilution water in the 6-well plate was removed without harming the larvae, and 3 mL of the test substance dilution at the corresponding concentration was immediately added to each well. After thorough mixing, the plate was covered and wrapped with aluminum foil, then incubated in a biochemical incubator at 28.5 °C ± 1.0 °C in the dark until the endpoint (4 dpf, total incubation time: 24 h).

Ten zebrafish with normal phenotype and behavior were randomly selected, fixed with 3% methylcellulose, and fluorescent images were captured under a microscope. Neutrophils were counted, and this index was used to evaluate the efficacy in improving sensitive skin (anti-inflammatory effect).

Statistical results are expressed as mean ± SD. *p* < 0.05 indicates a statistically significant difference.

## 3 Results

### 3.1 General toxicity

#### 3.1.1 Subchronic toxicity study (90-day feeding test)

##### 3.1.1.1 Clinical signs

During the entire experimental period, the activity, food intake, water consumption, and fecal characteristics of rats in all groups (both males and females) remained generally normal. No obvious behavioral changes, toxic signs, or animal deaths were observed.

##### 3.1.1.2 Body weight, food consumption, and food utilization

**Figure 1:**
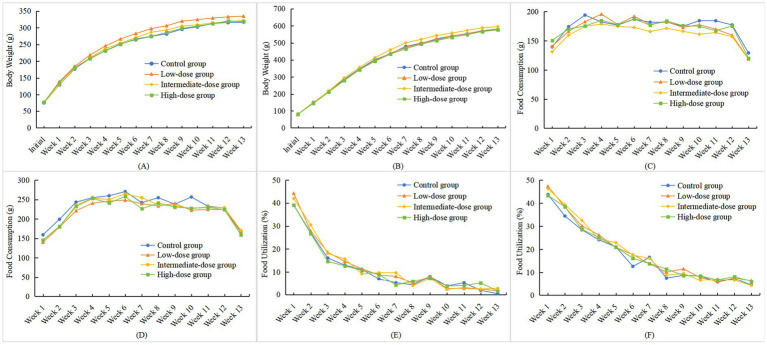
Effects of rhCol III on body weight, food intake, and food utilization rate in rats across each dose group. **(A)** Trend chart of body weight changes in female rats; **(B)** Trend chart of body weight changes in male rats; **(C)** Trend chart of food intake changes in female rats; **(D)** Trend chart of food intake changes in male rats; **(E)** Trend chart of food utilization rate changes in female rats; **(F)** Trend chart of food utilization rate changes in male rats (chart data are provided in Appendix A).

**Figure 2:**
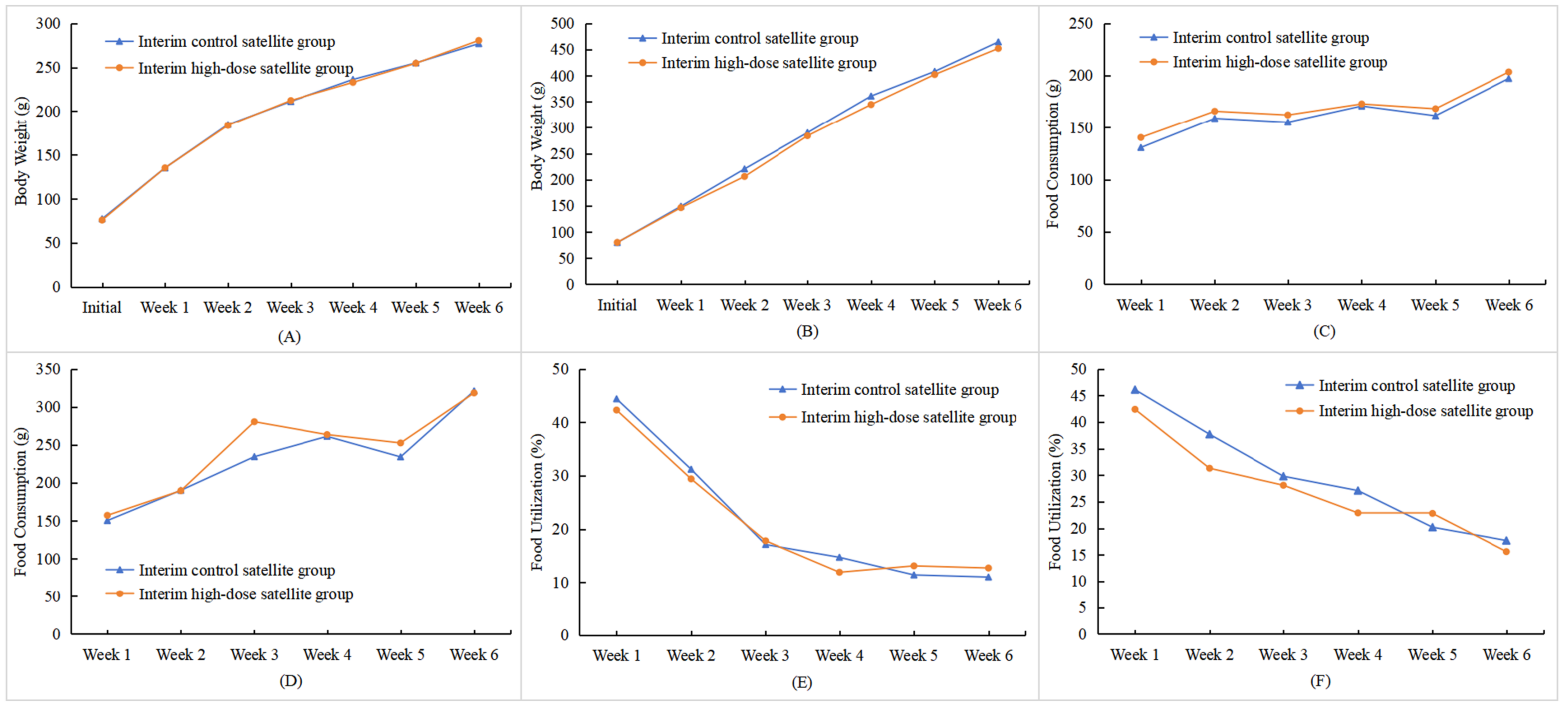
Effects of rhCol III on body weight, food intake, and food utilization rate in interim satellite group rats. **(A)** Trend chart of body weight changes in female rats; **(B)** trend chart of body weight changes in male rats; **(C)** trend chart of food intake changes in female rats; **(D)** trend chart of food intake changes in male rats; **(E)** trend chart of food utilization rate changes in female rats; **(F)** trend chart of food utilization rate changes in male rats (chart data are provided in Appendix A).

**Figure 3:**
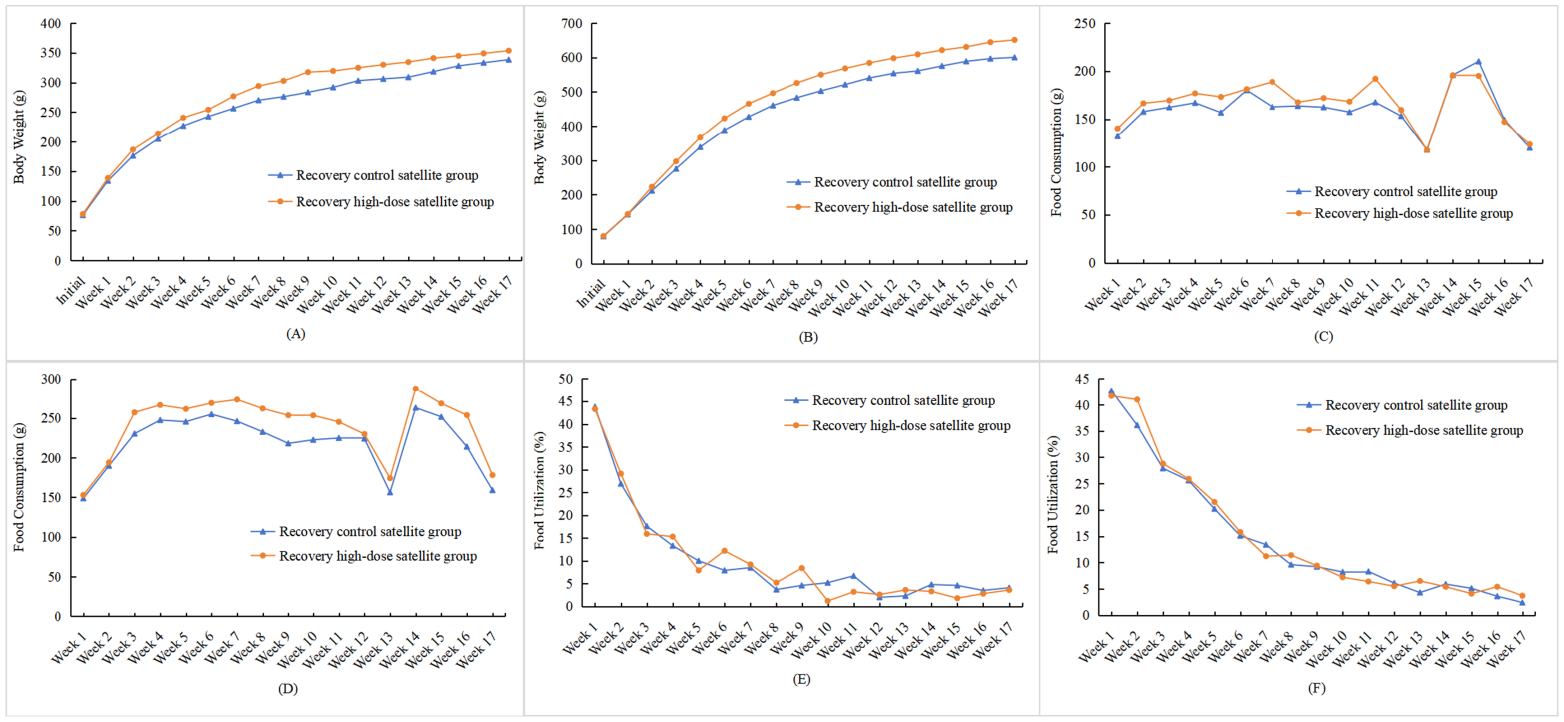
Effects of rhCol III on body weight, food intake, and food utilization rate in recovery satellite group rats. **(A)** Trend chart of body weight changes in female rats; **(B)** trend chart of body weight changes in male rats; **(C)** trend chart of food intake changes in female rats; **(D)** trend chart of food intake changes in male rats; **(E)** trend chart of food utilization rate changes in female rats; **(F)** trend chart of food utilization rate changes in male rats (chart data are provided in Appendix A).

As shown in Figures 1A,B, with the extension of the test duration, the body weights of rats in both the test group and the control group increased significantly, and their changing trends were generally consistent. Notably, the growth rate of female rats was lower than that of male rats. During the administration of the test sample, there were no statistically significant differences in body weights at each observation point between rats of both sexes in each dose group and those in the control group (*p* > 0.05). However, body weight in female rats of the low-dose group was higher than that in the control group at the end of Week 1 (*p* < 0.05); this isolated finding was not dose-dependent and did not persist, and was therefore considered an incidental fluctuation without toxicological significance. In the high-dose satellite group, no statistically significant differences in body weight at each observation point or in total weight gain were observed during either the interim satellite phase or the recovery period compared with the corresponding satellite control groups in either sex (*p* > 0.05) (Figures 2A,B, 3A,B). Analysis of variance results indicated that rhCol III had no significant effect on the body weight gain of rats.

As shown in Figures 1C,D, during the administration of the test sample, female rats in the high-dose group exhibited higher food intake than the control group in Week 1. Among male rats, the low-dose group showed lower food intake than the control group in Week 1 and Week 2, while the intermediate-dose group had lower food intake than the control group in Week 2. These differences were statistically significant (*p* < 0.05 or *p* < 0.01) but were considered normal fluctuations during the test period. For food intake at other time points and total food intake in male and female rats of all dose groups, no statistically significant differences were observed compared with the control group (*p* > 0.05).

During the satellite observations, food intake in male rats in the high-dose satellite group was higher than that in the corresponding satellite control group in Week 3 (*p* < 0.05) (Figures 2C,D). During the recovery period, statistically significant differences were observed at several isolated time points (e.g., Weeks 5 and 7 in females, and Weeks 3, 9, and 16 in males; *p* < 0.05 or *p* < 0.01) (Figures 3C,D). Because these changes were not sustained over time and showed no consistent dose–response pattern, they were interpreted as normal biological variation rather than treatment-related effects. No statistically significant differences were found in food intake at other time points or total food intake in male and female rats during the recovery period compared with the control satellite group (*p* > 0.05). Combined with the results of analysis of variance (ANOVA), it was indicated that rhCol III had no significant effect on the food intake of rats.

As shown in Figures 1E,F, during administration, the food utilization rate showed several statistically significant differences at isolated time points, including higher values in the intermediate-dose group at Weeks 2–3 in males and at Week 7 in females, and higher total food utilization in males in certain dose groups (*p* < 0.05 or *p* < 0.01). In the interim satellite phase, the food utilization rate in high-dose satellite males was lower than that in the satellite control at Week 4 (*p* < 0.05) (Figures 2E,F). During the recovery period, the food utilization rate in high-dose satellite females was lower than that in the recovery satellite control at Week 10 (*p* < 0.05) (Figures 3E,F). Additionally, apart from the time points noted above, no statistically significant differences (*p* > 0.05) were observed in food utilization at other observation time points in either sex compared with the control group.

Comprehensive analysis of full-cycle data revealed that the test sample had no persistent effect on the food utilization rate of either male or female rats. Although male rats showed statistically significant increases in food utilization rate at certain time points during the middle stage of the experiment and in total utilization rate, they were sporadic and did not show a consistent dose–response relationship. Such changes are presumably attributed to short-term fluctuations in feeding volume or metabolic status during the experiment, rather than substantial alterations in the organism’s energy utilization efficiency induced by the test sample. This result aligns with the stability of indicators such as body weight and food intake, thereby reinforcing the conclusion that the test sample does not exert a notable interference with the overall metabolism of rats.

##### 3.1.1.3 Ophthalmological examination

As shown in Table 1, all rats (males and females) in the control group and its satellite group, as well as in the low-, intermediate-, and high-dose groups and their corresponding satellite groups, exhibited normal findings on ophthalmological examination prior to dosing. As shown in Table 2, at the end of the study, ophthalmological examinations remained normal in both sexes in the control group and its satellite group and in the high-dose group and its satellite group. Overall, no treatment-related ocular abnormalities were observed throughout the study.

**Table 1:** Recombinant type III collagen 90-day oral toxicity study in rats: ophthalmological exam results at before dosing.

Sex	Test site	Dose groups			
		Control group (*n* = 20)	Low dose group (*n* = 10)	Intermediate-dose group (*n* = 10)	High dose group (*n* = 20)
Female	Cornea	0/20	0/10	0/10	0/20
	Lens	0/20	0/10	0/10	0/20
	Conjunctiva	0/20	0/10	0/10	0/20
	Iris	0/20	0/10	0/10	0/20
Male	Cornea	0/20	0/10	0/10	0/20
	Lens	0/20	0/10	0/10	0/20
	Conjunctiva	0/20	0/10	0/10	0/20
	Iris	0/20	0/10	0/10	0/20

Values are shown as number of animals with findings/total animals in group.

**Table 2:** Recombinant type III collagen 90-day oral toxicity study in rats: ophthalmological exam results at end of the interim period, end of dosing, and end of recovery.

Sex	Test items	Dose groups					
		End of the interim period		End of dosing		End of Recovery Period	
		Control satellite group (*n* = 5)	High-dose satellite group (*n* = 5)	Control group (*n* = 10)	High-dose group (*n* = 10)	Control satellite group (*n* = 5)	High-dose satellite group (*n* = 5)
Female	Cornea	0/5	0/5	0/10	0/10	0/5	0/5
	Lens	0/5	0/5	0/10	0/10	0/5	0/5
	Conjunctiva	0/5	0/5	0/10	0/10	0/5	0/5
	Iris	0/5	0/5	0/10	0/10	0/5	0/5
Male	Cornea	0/5	0/5	0/10	0/10	0/5	0/5
	Lens	0/5	0/5	0/10	0/10	0/5	0/5
	Conjunctiva	0/5	0/5	0/10	0/10	0/5	0/5
	Iris	0/5	0/5	0/10	0/10	0/5	0/5

Values are shown as number of animals with findings/total animals in group.

##### 3.1.1.4 Hematological indicators, blood biochemical indicators, and urinalysis indicators

According to the data shown in Table 3, most hematological indices, including total white blood cell count and differential (NEU, LYM, MON, BAS), red blood cell parameters (RBC, HGB, HCT), platelet parameters (PLT), and coagulation function indices (PT, APTT) in rats of both sexes across all dose groups showed no statistically significant differences compared with the control group (*p* > 0.05). Only the eosinophil percentage (EOS%) in female rats of the low-dose group was 0.83 ± 0.63%, which was significantly lower than that in the control group (2.11 ± 0.99%) (*p* < 0.05). However, this difference exhibited no dose–response relationship (no differences were observed between the intermediate- and high-dose groups and the control group), and the animals did not present associated manifestations such as allergic reactions or parasitic infections. Combined with the stability of other hematological indices, this discrepancy was speculated to reflect individual variability during the experiment rather than biological toxicity.

**Table 3:** Statistical summary table of animal hematological indices for the test group in the 90-day oral toxicity test in rats with recombinant type III collagen ( $\bar{x}$ ± s).

Sex	Test items	Dose groups (*n* = 10)			
		Control group	Low dose group	Intermediate-dose group	High dose group
Female	WBC (×10^9^/L)	3.00 ± 0.68	3.37 ± 0.86	2.83 ± 0.84	3.02 ± 0.69
	NEU (%)	14.32 ± 4.04	13.78 ± 5.90	14.25 ± 2.88	12.44 ± 2.77
	LYM (%)	80.39 ± 4.18	80.92 ± 6.83	78.81 ± 4.36	81.44 ± 4.15
	MON (%)	3.18 ± 1.46	4.45 ± 1.93	4.34 ± 2.04	3.86 ± 2.30
	EOS (%)	2.11 ± 0.99	0.83 ± 0.63*	2.60 ± 1.82	2.26 ± 1.24
	BAS (%)	0.00 ± 0.00	0.02 ± 0.06	0.00 ± 0.00	0.00 ± 0.00
	RBC (×10^12^/L)	7.64 ± 0.58	7.74 ± 0.63	7.69 ± 0.54	8.06 ± 0.95
	HGB (g/L)	154.00 ± 9.30	158.10 ± 10.60	156.30 ± 8.40	161.40 ± 16.30
	HCT (%)	43.30 ± 3.00	44.40 ± 3.60	44.0 ± 2.6	45.70 ± 5.40
	PLT (×10^9^/L)	775.00 ± 269.00	724.00 ± 239.00	885.00 ± 105.00	874.00 ± 209.00
	PT (sec)	14.90 ± 0.90	15.00 ± 0.80	14.50 ± 0.60	15.00 ± 1.20
	APTT (sec)	15.50 ± 2.20	14.90 ± 1.40	14.30 ± 1.00	15.90 ± 3.70
Male	WBC (×10^9^/L)	4.40 ± 0.76	4.55 ± 1.36	4.38 ± 1.38	4.39 ± 1.35
	NEU (%)	16.08 ± 5.37	18.47 ± 3.84	15.65 ± 3.99	19.46 ± 3.68
	LYM (%)	78.62 ± 7.16	76.59 ± 3.97	78.79 ± 5.31	74.84 ± 3.43
	MON (%)	3.72 ± 1.74	3.33 ± 0.58	3.87 ± 1.47	4.26 ± 1.49
	EOS (%)	1.58 ± 0.78	1.61 ± 0.74	1.68 ± 0.79	1.44 ± 0.65
	BAS (%)	0.00 ± 0.00	0.00 ± 0.00	0.01 ± 0.03	0.00 ± 0.00
	RBC (×10^12^/L)	8.33 ± 0.77	8.37 ± 0.77	8.62 ± 0.59	8.34 ± 0.43
	HGB (g/L)	161.10 ± 8.9	165.40 ± 14.5	166.70 ± 7.90	160.00 ± 9.80
	HCT (%)	45.30 ± 2.40	46.7 ± 4.3	46.70 ± 2.40	45.70 ± 2.70
	PLT (×10^9^/L)	765.00 ± 78.00	779.00 ± 93.00	826.00 ± 128.00	796.00 ± 98.00
	PT (sec)	15.90 ± 0.40	15.70 ± 0.60	15.80 ± 0.70	15.90 ± 0.40
	APTT (sec)	17.80 ± 1.30	17.80 ± 1.40	17.70 ± 1.90	17.50 ± 1.00

*Indicates a statistically significant difference compared with the control group (*p* < 0.05); *n* represents the number of animals per group.

The data in Table 4 further confirmed the above conclusions: at both the interim assessment and the end of the recovery period, all hematological indices in rats in the high-dose satellite group (both sexes) showed no statistically significant differences compared with the control satellite group (*p* > 0.05).

**Table 4:** Statistical summary table of animal hematological indices for the satellite group in the 90-day oral toxicity test in rats with Recombinant type III collagen ( $\bar{x}$ ± s).

Sex	Test items	Dose groups (*n* = 5)			
		Interim period		End of recovery period	
		Control satellite group	High-dose satellite group	Control satellite group	High-dose satellite group
Female	WBC (×10^9^/L)	3.41 ± 0.44	3.75 ± 1.25	2.45 ± 0.55	2.50 ± 0.97
	NEU (%)	13.02 ± 4.01	14.78 ± 6.96	11.14 ± 5.80	11.42 ± 4.88
	LYM (%)	80.58 ± 4.20	80.34 ± 7.93	82.60 ± 5.22	78.56 ± 2.67
	MON (%)	4.58 ± 2.30	3.04 ± 1.25	4.32 ± 1.47	5.88 ± 2.03
	EOS (%)	1.82 ± 0.38	1.84 ± 1.15	1.94 ± 0.31	4.10 ± 4.43
	BAS (%)	0.00 ± 0.00	0.00 ± 0.00	0.00 ± 0.00	0.04 ± 0.09
	RBC (×10^12^/L)	7.05 ± 0.49	7.08 ± 0.20	7.27 ± 0.67	7.32 ± 0.54
	HGB (g/L)	145.80 ± 8.60	148.60 ± 5.00	143.00 ± 8.20	143.00 ± 4.70
	HCT (%)	42.20 ± 2.60	43.00 ± 1.20	40.00 ± 1.80	40.30 ± 1.50
	PLT (×10^9^/L)	934.00 ± 86.00	941.00 ± 205.00	839.00 ± 80.00	701.00 ± 214.00
	PT (sec)	14.20 ± 0.70	14.00 ± 0.40	14.10 ± 0.40	14.80 ± 0.80
	APTT (sec)	12.80 ± 1.30	13.40 ± 0.80	14.00 ± 0.90	14.40 ± 0.90
Male	WBC (×10^9^/L)	5.65 ± 2.09	5.42 ± 0.75	6.89 ± 2.61	7.38 ± 1.86
	NEU (%)	16.22 ± 5.01	13.82 ± 2.97	16.76 ± 4.21	12.56 ± 3.18
	LYM (%)	79.52 ± 5.37	81.82 ± 3.52	76.82 ± 4.41	81.02 ± 4.26
	MON (%)	2.96 ± 1.26	3.10 ± 0.51	4.38 ± 0.67	4.28 ± 1.04
	EOS (%)	1.28 ± 0.47	1.26 ± 0.27	1.90 ± 0.70	2.14 ± 1.09
	BAS (%)	0.02 ± 0.04	0.00 ± 0.00	0.14 ± 0.31	0.00 ± 0.00
	RBC (×10^12^/L)	7.38 ± 0.29	7.65 ± 0.22	8.46 ± 0.34	8.26 ± 0.44
	HGB (g/L)	155.60 ± 6.70	156.40 ± 5.30	156.20 ± 6.50	153.20 ± 9.40
	HCT (%)	46.70 ± 1.60	47.00 ± 1.50	45.10 ± 1.70	44.50 ± 3.10
	PLT (×10^9^/L)	1134.00 ± 177.00	981.00 ± 90.00	934.00 ± 159.00	847.00 ± 35.00
	PT (sec)	13.30 ± 0.80	14.20 ± 1.10	16.60 ± 1.20	17.10 ± 1.60
	APTT (sec)	17.70 ± 1.30	19.10 ± 3.60	17.50 ± 0.60	17.20 ± 0.30

*n* represents the number of animals per group.

Overall, 90-day oral administration of rhCol III did not induce biologically significant abnormal changes in the hematological indices of rats. This supports the conclusion that the test substance has no cumulative or persistent effects on the rat hematological system, indicating its safety for the rat hematological system at the tested doses.

The data in Table 5 indicated that at the end of the trial, only the K^+^ concentration in female rats of the low-dose group (3.47 ± 0.21 mmol/L) was statistically significantly lower than that in the control group (3.89 ± 0.19 mmol/L) (*p* < 0.05). However, this difference exhibited no dose–response relationship, and no clinical toxic manifestations related to electrolyte imbalance were observed in the animals. For all other hematobiochemical indices, including total protein (TP), albumin (Alb), hepatic enzymes (AST, ALT, ALP, GGT), blood lipids (TC, TG), renal function indicators (Urea, Cr), blood glucose (Glu), and electrolytes (Cl^−^, Na^+^), no statistically significant differences were detected between any dose group (males or females) and the control group (*p* > 0.05), suggesting that the test substance did not induce systematic alterations in the hematobiochemical profiles of rats.

**Table 5:** Statistical summary table of animal hematobiochemical indices for the test group in the 90-day oral toxicity test in rats with recombinant type III collagen ( $\bar{x}$ ± s).

Sex	Test items	Dose groups (*n* = 10)			
		Control group	Low dose group	Intermediate-dose group	High dose group
Female	TP (g/L)	70.60 ± 5.30	67.30 ± 3.90	70.40 ± 4.80	69.80 ± 5.20
	Alb (g/L)	42.10 ± 3.00	40.20 ± 2.80	42.10 ± 2.80	42.30 ± 3.00
	AST (U/L)	185.20 ± 159.40	140.10 ± 55.70	155.00 ± 111.60	141.20 ± 61.50
	ALT (U/L)	56.90 ± 49.90	45.80 ± 21.10	46.90 ± 33.20	48.60 ± 27.80
	ALP (U/L)	57.60 ± 13.80	57.30 ± 16.80	58.40 ± 12.20	47.20 ± 11.40
	GGT (U/L)	0.56 ± 0.34	0.68 ± 0.54	0.55 ± 0.41	0.45 ± 0.22
	TC (mmol/L)	2.93 ± 0.82	2.75 ± 0.48	2.94 ± 0.32	2.68 ± 0.45
	TG (mmol/L)	0.54 ± 0.33	0.53 ± 0.24	0.61 ± 0.28	0.52 ± 0.18
	Urea (mmol/L)	8.91 ± 0.75	8.37 ± 1.41	9.19 ± 1.04	8.52 ± 0.74
	Cr (μmol/L)	52.40 ± 6.70	55.80 ± 5.70	54.90 ± 7.60	51.40 ± 7.30
	Glu (mmol/L)	9.26 ± 0.58	8.32 ± 1.13	8.95 ± 1.13	8.30 ± 1.38
	K^+^ (mmol/L)	3.89 ± 0.19	3.47 ± 0.21*	3.77 ± 0.27	3.63 ± 0.46
	Cl^−^ (mmol/L)	107.90 ± 1.30	108.00 ± 1.20	107.30 ± 1.40	107.90 ± 0.60
	Na^+^ (mmol/L)	140.90 ± 1.80	142.00 ± 1.40	140.90 ± 1.40	141.60 ± 1.80
Male	TP (g/L)	58.80 ± 1.20	58.30 ± 1.40	58.60 ± 2.40	56.80 ± 1.60
	Alb (g/L)	34.10 ± 0.80	33.50 ± 0.70	33.70 ± 1.30	33.20 ± 0.90
	AST (U/L)	112.80 ± 22.90	110.80 ± 13.60	111.10 ± 23.30	118.90 ± 20.60
	ALT (U/L)	33.10 ± 7.50	31.80 ± 6.60	32.80 ± 7.20	34.10 ± 10.00
	ALP (U/L)	104.10 ± 15.20	97.70 ± 10.40	97.40 ± 19.70	111.80 ± 20.90
	GGT (U/L)	0.64 ± 0.13	0.64 ± 0.14	0.60 ± 0.21	0.71 ± 0.10
	TC (mmol/L)	1.86 ± 0.30	2.03 ± 0.34	2.02 ± 0.37	1.71 ± 0.21
	TG (mmol/L)	0.56 ± 0.28	0.39 ± 0.16	0.68 ± 0.66	0.63 ± 0.71
	Urea (mmol/L)	6.44 ± 0.91	6.84 ± 0.68	6.78 ± 0.63	6.75 ± 0.88
	Cr (μmol/L)	44.40 ± 4.30	47.20 ± 7.20	49.40 ± 2.90	47.60 ± 2.90
	Glu (mmol/L)	9.23 ± 1.10	9.62 ± 1.55	10.89 ± 1.93	10.19 ± 3.68
	K^+^ (mmol/L)	4.42 ± 0.26	4.31 ± 0.28	4.40 ± 0.24	4.40 ± 0.14
	Cl^−^ (mmol/L)	108.90 ± 1.30	109.10 ± 1.90	107.50 ± 1.30	108.60 ± 1.30
	Na^+^ (mmol/L)	141.40 ± 2.30	142.00 ± 1.50	141.10 ± 1.00	141.60 ± 1.10

*Indicates a statistically significant difference compared with the control group (*p* < 0.05); *n*: represents the number of animals per group.

The data in Table 6 further supported these conclusions. During the interim treatment period, the creatinine concentration (Cr) in female rats of the high-dose satellite group (37.2 ± 2.1 μmol/L) was significantly lower than that in the control satellite group (42.0 ± 2.8 μmol/L) (*p* < 0.05). The mild reduction in K^+^ in low-dose group females and the transient decrease in Cr in high-dose satellite group females were isolated differences with no dose dependence or toxicological association, primarily attributed to random experimental variability rather than toxic effects of the test substance. No statistically significant differences were observed for any other hematobiochemical indices between the high-dose satellite group and the control satellite group at the interim assessment or at the end of the recovery period in either sex (*p* > 0.05), indicating no persistent or delayed abnormalities after cessation of administration.

**Table 6:** Statistical summary table of animal hematobiochemical indices for the satellite group in the 90-day oral toxicity test in rats with recombinant type III collagen ( $\bar{x}$ ± s).

Sex	Test items	Dose groups (*n* = 5)			
		Interim period		End of recovery period	
		Control satellite group	High-dose satellite group	Control satellite group	High-dose satellite group
Female	TP (g/L)	60.50 ± 3.30	59.20 ± 2.60	64.60 ± 3.40	68.80 ± 4.20
	Alb (g/L)	36.10 ± 2.30	35.30 ± 0.60	39.90 ± 2.90	42.70 ± 2.30
	AST (U/L)	95.50 ± 14.20	83.50 ± 4.40	111.10 ± 31.00	109.30 ± 41.00
	ALT (U/L)	24.70 ± 1.40	24.80 ± 1.10	42.50 ± 10.00	42.60 ± 15.00
	ALP (U/L)	106.40 ± 22.70	102.60 ± 19.00	49.90 ± 12.10	55.30 ± 23.40
	GGT (U/L)	0.52 ± 0.30	0.86 ± 0.18	0.80 ± 0.12	0.74 ± 0.27
	TC (mmol/L)	2.34 ± 0.51	2.57 ± 0.39	2.42 ± 0.52	2.77 ± 0.58
	TG (mmol/L)	0.34 ± 0.13	0.40 ± 0.07	0.49 ± 0.13	1.05 ± 0.54
	Urea (mmol/L)	9.22 ± 0.78	8.80 ± 0.54	8.83 ± 0.67	7.94 ± 0.46
	Cr (μmol/L)	42.00 ± 2.80	37.20 ± 2.10*	50.60 ± 6.20	48.20 ± 3.30
	Glu (mmol/L)	7.11 ± 0.70	7.17 ± 1.54	7.69 ± 1.19	8.74 ± 1.09
	K^+^ (mmol/L)	3.73 ± 0.31	3.97 ± 0.31	3.82 ± 0.20	3.77 ± 0.11
	Cl^−^ (mmol / L)	105.40 ± 0.30	105.90 ± 0.60	107.50 ± 0.90	107.60 ± 0.60
	Na^+^ (mmol/L)	135.40 ± 1.10	136.70 ± 0.80	139.20 ± 1.20	139.30 ± 1.00
Male	TP (g/L)	57.40 ± 1.40	55.80 ± 2.20	56.10 ± 1.70	57.00 ± 2.10
	Alb (g/L)	33.40 ± 0.80	33.00 ± 1.30	34.00 ± 1.10	34.60 ± 0.30
	AST (U/L)	101.70 ± 8.10	93.00 ± 6.10	120.70 ± 22.70	104.60 ± 24.30
	ALT (U/L)	34.00 ± 4.20	33.50 ± 5.00	42.60 ± 13.70	32.70 ± 6.20
	ALP (U/L)	197.00 ± 25.90	212.60 ± 28.10	91.40 ± 13.50	93.70 ± 21.30
	GGT (U/L)	0.52 ± 0.41	0.54 ± 0.36	0.88 ± 0.27	0.86 ± 0.23
	TC (mmol/L)	1.94 ± 0.17	1.83 ± 0.19	1.78 ± 0.33	1.94 ± 0.40
	TG (mmol/L)	0.72 ± 0.30	0.43 ± 0.30	0.38 ± 0.09	0.76 ± 0.53
	Urea (mmol/L)	6.74 ± 0.67	6.55 ± 0.69	6.68 ± 0.30	6.25 ± 0.84
	Cr (μmol/L)	34.70 ± 3.60	36.00 ± 2.70	56.10 ± 5.40	53.50 ± 10.90
	Glu (mmol/L)	8.71 ± 0.87	7.32 ± 1.03	9.68 ± 1.85	11.39 ± 2.80
	K^+^ (mmol/L)	4.38 ± 0.07	4.20 ± 0.17	4.40 ± 0.40	4.48 ± 0.16
	Cl^−^ (mmol / L)	105.90 ± 0.70	106.20 ± 0.70	106.40 ± 0.30	105.50 ± 0.90
	Na^+^ (mmol/L)	135.90 ± 0.70	136.70 ± 0.30	139.60 ± 0.60	138.20 ± 1.20

*Indicates a statistically significant difference compared with the control group (*p* < 0.05); *n*: represents the number of animals per group.

Collectively, the results from the main test and satellite groups demonstrated that 90-day oral administration of rhCol III did not induce biologically significant abnormal changes in the hematobiochemical indices of rats. The overall stability of all indices further supports the conclusion that the test substance has no adverse effects on the rat hematobiochemical system at the tested doses.

The data in Table 7 showed that at the end of the trial, there were no statistically significant differences (*p* > 0.05) in urinary indices between rats of all dose groups (both males and females) and the control group. Specifically, for female rats, the detection rates of LEU (0/10 to 1/10) and KET (0/10 to 1/10) in all dose groups showed no significant differences compared to the control group (LEU 0/10, KET 0/10). For male rats, the detection rates of LEU (0/10), PRO (1/10 to 2/10), and KET (1/10 to 3/10) in all dose groups showed no significant differences compared to the control group (LEU 0/10, PRO 1/10, KET 1/10), and the detection rates of other indices were consistent with those in the control group.

**Table 7:** Statistical summary table of animal urinary indices for the test group in the 90-day oral toxicity test in rats with recombinant type III collagen ( $\bar{x}$ ± s).

Sex	Test items	Dose groups (*n* = 10)			
		Control group	Low dose group	Intermediate-dose group	High dose group
Female	SG	1.001 ± 0.002	1.002 ± 0.002	1.001 ± 0.002	1.000 ± 0.002
	LEU	0/10	1/10	0/10	0/10
	PH	8.9 ± 0.3	8.8 ± 0.4	9.0 ± 0.0	9.0 ± 0.0
	ERY	0/10	0/10	0/10	0/10
	PRO	0/10	0/10	0/10	0/10
	GLU	0/10	0/10	0/10	0/10
	ASC	0/10	0/10	0/10	0/10
	KET	0/10	1/10	1/10	1/10
	UBG	0/10	0/10	0/10	0/10
	BIL	0/10	0/10	0/10	0/10
Male	SG	1.005 ± 0.002	1.002 ± 0.003	1.004 ± 0.008	1.003 ± 0.004
	LEU	0/10	0/10	0/10	0/10
	PH	8.6 ± 0.8	8.8 ± 0.4	8.6 ± 1.0	8.6 ± 0.7
	ERY	0/10	0/10	0/10	0/10
	PRO	1/10	2/10	1/10	2/10
	GLU	0/10	0/10	0/10	0/10
	ASC	0/10	0/10	0/10	1/10
	KET	1/10	3/10	1/10	1/10
	UBG	0/10	0/10	0/10	0/10
	BIL	0/10	0/10	0/10	0/10

The data shown in columns such as test items GLU, PRO, BIL, KET represent the number of positive animals / number of animals examined; *n* represents the number of animals per group.

The data in Table 8 further validated these conclusions: at both the interim treatment period and the end of the recovery period, there were no statistically significant differences (*p* > 0.05) in urinary indices between high-dose satellite group rats (both males and females) and the control satellite group.

**Table 8:** Statistical summary table of animal urinary indices for the satellite group in the 90-day oral toxicity test in rats with recombinant type III collagen ( $\bar{x}$ ± s).

Sex	Test items	Dose groups (*n* = 5)			
		Interim period		End of recovery period	
		Control satellite group	High-dose satellite group	Control satellite group	High-dose satellite group
Female	SG	1.000 ± 0.000	1.001 ± 0.002	1.001 ± 0.002	1.000 ± 0.000
	LEU	0/5	0/5	0/5	1/5
	PH	9.0 ± 0.0	9.0 ± 0.0	9.0 ± 0.0	9.0 ± 0.0
	ERY	0/5	0/5	0/5	0/5
	PRO	0/5	0/5	0/5	0/5
	GLU	0/5	0/5	0/5	0/5
	ASC	0/5	0/5	0/5	0/5
	KET	0/5	0/5	0/5	0/5
	UBG	0/5	0/5	0/5	0/5
	BIL	0/5	0/5	0/5	0/5
Male	SG	1.004 ± 0.002	1.002 ± 0.003	1.003 ± 0.003	1.005 ± 0.004
	LEU	0/5	1/5	0/5	2/5
	PH	9.0 ± 0.00	8.8 ± 0.4	9.0 ± 0.0	8.6 ± 0.8
	ERY	0/5	0/5	0/5	0/5
	PRO	0/5	0/5	0/5	0/5
	GLU	0/5	0/5	0/5	0/5
	ASC	0/5	0/5	0/5	0/5
	KET	1/5	0/5	1/5	0/5
	UBG	0/5	0/5	0/5	0/5
	BIL	0/5	0/5	0/5	0/5

The data shown in columns such as test items GLU, PRO, BIL, KET represent the number of positive animals / number of animals examined; *n* represents the number of animals per group.

Combining the results from the main test and satellite groups, 90-day oral administration of rhCol III did not induce statistically significant abnormal changes in the urinary indices of rats. Although some inter-group differences were observed in certain indices, these were attributed to random individual variability or detection errors rather than toxicological effects, as they lacked statistical significance and were not associated with clinical symptoms such as urinary system irritation or renal dysfunction. The overall stability of all indices further supports that rhCol III has no significant impact on the rat urinary system, reinforcing its safety at the tested doses.

##### 3.1.1.5 Necropsy and organ weight

The data in Tables 9, 10 show that at the end of the necropsy and organ weight trial, there were no statistically significant differences (*p* > 0.05) in organ weights or organ-to-body weight ratios between rats of all dose groups (both males and females) and the control group. This indicates that rhCol III did not induce cardiac hypertrophy or atrophy, nor did it cause any gross structural changes. The liver weights of all dose group rats were generally consistent with those of the control group; combined with the stability of blood biochemical indices (e.g., ALT, AST), this further ruled out the possibility of hepatic toxicity or damage. The thymus weights of rats in all dose group also showed no differences from the control group, consistent with the stability of immune function-related indices (e.g., lymphocyte proportion), indicating that collagen did not interfere with immune system development.

**Table 9:** Statistical summary table of animal organ weights for the test group in the 90-day oral toxicity test in rats with recombinant type III collagen (g, $\bar{x}$ ± s).

Sex	Test items	Dose groups (*n* = 10)			
		Control group	Low dose group	Intermediate-dose group	High dose group
Female	Body weight after fasting	297.6 ± 37.5	317.3 ± 25.1	305.0 ± 25.3	303.5 ± 35.8
	Heart	0.958 ± 0.146	0.977 ± 0.138	0.942 ± 0.096	0.921 ± 0.096
	Thymus	0.439 ± 0.112	0.389 ± 0.109	0.413 ± 0.089	0.354 ± 0.104
	Liver	8.785 ± 1.414	8.597 ± 0.726	8.326 ± 0.762	8.683 ± 1.654
	Spleen	0.613 ± 0.098	0.548 ± 0.085	0.579 ± 0.077	0.540 ± 0.096
	Kidney	1.977 ± 0.260	1.948 ± 0.087	1.917 ± 0.150	1.900 ± 0.262
	Adrenal gland	0.070 ± 0.014	0.063 ± 0.015	0.070 ± 0.019	0.060 ± 0.015
	Brain	1.993 ± 0.108	2.036 ± 0.119	2015 ± 0.096	2.010 ± 0.175
	Ovary	0.133 ± 0.029	0.118 ± 00.31	0.142 ± 0.017	0.116 ± 0.025
	Uterus	1.160 ± 0.632	0.786 ± 0.140	0.986 ± 0.257	0.862 ± 0.293
Male	Body weight after fasting	551.9 ± 31.6	551.9 ± 61.3	569.5 ± 49.6	549.5 ± 43.9
	Heart	1.572 ± 0.216	1.576 ± 0.226	1.550 ± 0.143	1.684 ± 0.217
	Thymus	0.527 ± 0.074	0.495 ± 0.093	0.481 ± 0.103	0.579 ± 0.163
	Liver	14.303 ± 1.726	14.294 ± 2.452	14.914 ± 2.202	14.260 ± 2.220
	Spleen	0.933 ± 0.136	0.998 ± 0.162	0.898 ± 0.158	1.044 ± 0.183
	Kidney	3.313 ± 0.302	3.415 ± 0.458	3.251 ± 0.211	3.219 ± 0.384
	Adrenal gland	0.067 ± 0.016	0.076 ± 0.019	0.074 ± 0.016	0.073 ± 0.018
	Brain	2.203 ± 0.076	2.195 ± 0.036	2.185 ± 0.096	2.199 ± 0.103
	Testis	4.010 ± 0.367	3.916 ± 0.301	4.320 ± 0.515	4.017 ± 0.189
	Epididymis	1.640 ± 0.137	1.622 ± 0.219	1.653 ± 0.199	1.693 ± 0.276

*n* represents the number of animals per group.

**Table 10:** Statistical summary table of animal organ coefficients for the test group in the 90-day oral toxicity test in rats with recombinant type III collagen ( $\bar{x}$ ± s).

Sex	Test items	Dose groups (*n* = 10)			
		Control group	Low dose group	Intermediate-dose group	High dose group
Female	Heart/body	0.322 ± 0.027	0.311 ± 0.041	0.309 ± 0.040	0.304 ± 0.032
	Thymus/body	0.146 ± 0.026	0.124 ± 0.037	0.135 ± 0.026	0.116 ± 0.030
	Liver/body	2.941 ± 0.177	2.713 ± 0.174	2.731 ± 0.169	2.843 ± 0.276
	Spleen/body	0.209 ± 0.045	0.174 ± 0.028	0.191 ± 0.025	0.178 ± 0.022
	Kidney/body	0.665 ± 0.043	0.617 ± 0.046	0.630 ± 0.041	0.626 ± 0.048
	Adrenal gland/body	0.024 ± 0.004	0.020 ± 0.005	0.023 ± 0.006	0.020 ± 0.004
	Brain/body	0.677 ± 0.070	0.646 ± 0.066	0.664 ± 0.050	0.667 ± 0.064
	Ovary/body	0.045 ± 0.010	0.038 ± 0.011	0.047 ± 0.005	0.039 ± 0.009
	Uterus/body	0.394 ± 0.206	0.249 ± 0.046	0.326 ± 0.093	0.288 ± 0.103
Male	Heart/body	0.283 ± 0.036	0.284 ± 0.021	0.272 ± 0.022	0.306 ± 0.036
	Thymus/body	0.096 ± 0.012	0.090 ± 0.014	0.083 ± 0.017	0.105 ± 0.025
	Liver/body	2.587 ± 0.227	2.576 ± 0.211	2.612 ± 0.231	2.586 ± 0.266
	Spleen/body	0.170 ± 0.021	0.179 ± 0.018	0.157 ± 0.018	0.190 ± 0.025
	Kidney/body	0.600 ± 0.045	0.620 ± 0.058	0.573 ± 0.040	0.584 ± 0.048
	Adrenal gland/body	0.012 ± 0.002	0.014 ± 0.004	0.013 ± 0.002	0.013 ± 0.003
	Brain/body	0.401 ± 0.030	0.402 ± 0.046	0.386 ± 0.034	0.402 ± 0.025
	Testis/body	0.727 ± 0.059	0.718 ± 0.103	0.764 ± 0.122	0.735 ± 0.065
	Epididymis/body	0.298 ± 0.028	0.296 ± 0.045	0.292 ± 0.038	0.309 ± 0.046

*n* represents the number of animals per group.

Furthermore, the organ weights and organ-to-body weight ratios of high-dose satellite group rats (both males and females) showed no statistically significant differences compared to the control satellite group (*p* > 0.05) (Tables 11, 12), which validated these conclusions moreover.

**Table 11:** Statistical summary table of animal organ weights for the satellite group in the 90-day oral toxicity test in rats with recombinant type III collagen (g, $\bar{x}$ ± s).

Sex	Test items	Dose groups (*n* = 5)	
		Recovery period control satellite group	Recovery period high-dose satellite group
Female	Body weight after fasting	311.9 ± 26.9	335.3 ± 42.8
	Heart	1.120 ± 0.080	1.100 ± 0.029
	Thymus	0.276 ± 0.063	0.268 ± 0.083
	Liver	8.470 ± 0.992	8.749 ± 0.728
	Spleen	0.503 ± 0.069	0.565 ± 0.087
	Kidney	1.880 ± 0.165	1.877 ± 0.109
	Adrenal gland	0.059 ± 0.009	0.056 ± 0.009
	Brain	1.981 ± 0.076	1.992 ± 0.073
	Ovary	0.139 ± 0.018	0.133 ± 0.023
	Uterus	0.851 ± 0.184	0.935 ± 0.213
Male	Body weight after fasting	576.0 ± 60.9	621.3 ± 51.2
	Heart	1.569 ± 0.088	1.701 ± 0.121
	Thymus	0.352 ± 0.088	0.480 ± 0.133
	Liver	13.029 ± 1.459	14.563 ± 1.132
	Spleen	0.872 ± 0.148	0.994 ± 0.057
	Kidney	3.113 ± 0.319	3.422 ± 0.133
	Adrenal gland	0.055 ± 0.009	0.067 ± 0.012
	Brain	2.160 ± 0.066	2.236 ± 0.166
	Testis	4.197 ± 0.286	4.347 ± 0.468
	Epididymis	1.686 ± 0.297	1.803 ± 0.275

**Table 12:** Statistical summary table of animal organ coefficients for the satellite group in the 90-day oral toxicity test in rats with recombinant type III collagen ( $\bar{x}$ ± s).

Sex	Test items	Dose groups (*n* = 5)	
		Recovery Period control satellite group	Recovery period high-dose satellite group
Female	Heart/body	0.360 ± 0.034	0.334 ± 0.039
	Thymus/body	0.086 ± 0.011	0.080 ± 0.026
	Liver/body	2.734 ± 0.382	2.636 ± 0.300
	Spleen/body	0.162 ± 0.022	0.170 ± 0.032
	Kidney/body	0.606 ± 0.063	0.566 ± 0.058
	Adrenal gland/body	0.019 ± 0.003	0.017 ± 0.004
	Brain/body	0.637 ± 0.036	0.600 ± 0.063
	Ovary/body	0.045 ± 0.007	0.040 ± 0.005
	Uterus/body	0.278 ± 0.075	0.281 ± 0.066
Male	Heart/body	0.276 ± 0.029	0.272 ± 0.013
	Thymus/body	0.060 ± 0.020	0.078 ± 0.020
	Liver/body	2.262 ± 0.149	2.346 ± 0.130
	Spleen/body	0.152 ± 0.037	0.162 ± 0.013
	Kidney/body	0.544 ± 0.064	0.552 ± 0.031
	Adrenal gland/body	0.010 ± 0.002	0.011 ± 0.001
	Brain/body	0.378 ± 0.037	0.361 ± 0.032
	Testis/body	0.735 ± 0.097	0.700 ± 0.041
	Epididymis/body	0.294 ± 0.057	0.290 ± 0.036

##### 3.1.1.6 Histopathology

After the experiment, all experimental animals underwent gross dissection, and no abnormal changes were observed visually. Further histopathological examinations were conducted on multiple organs (covering 21 key organs, including the brain, pituitary, thyroid gland, thymus, heart, liver, spleen, kidney, adrenal gland, digestive system, lymphatic system, urinary system, reproductive system, and respiratory system) in both the control group and high-dose group animals. The examination results showed that, except for 2 cases of mild inflammatory cell infiltration in the prostate glands of male rats in the control group, no sample-related pathological lesions were observed in any other organs (including tissues from the high-dose group and other tissues in the control group). Relevant data are provided in Appendix B.

Based on all histopathological results, 90-day oral administration of rhCol III did not induce any sample-related pathological changes in the organ systems of rats. The mild inflammatory infiltration in the prostate glands represents a spontaneous lesion (e.g., non-specific bacterial infection or minor mechanical stimulation) in the animals and does not affect the overall evaluation of the sample’s safety. This further provides reliable histopathological evidence for the safety of rhCol III at the tested dosage in terms of organ tissue safety.

#### 3.1.2 Genotoxicity studies

##### 3.1.2.1 Bacterial reverse mutation assay (Ames test)

In both the main and validation tests, no precipitation of the test substance was observed in any dose group after the addition of the test solution, regardless of the presence or absence of the metabolic activation system (+S9/−S9). Moreover, at the end of the incubation period, the background bacterial lawn of each tested strain showed no significant difference compared to the untreated control group, indicating that the test substance exhibited no bacterial toxicity at doses up to 5,000 μg/plate. As shown in Tables 13, 14, under both metabolic activation conditions, the number of revertant colonies in all dose groups did not exceed twice that of the untreated control group across the five tested strains. In contrast, the mean number of revertant colonies in all positive control groups reached more than twice that of the untreated control group, demonstrating a clear positive response (Tables 15, 16). In summary, rhCol III did not induce an increase in revertant colony numbers in the five tested strains, and the Ames test result was negative.

**Table 13:** Results of bacterial reverse mutation test with recombinant type III collagen (main test).

Group		Dose (/per plate)	TA97a		TA98		TA100		TA102		TA1535	
			−S9	+S9	−S9	+S9	−S9	+S9	−S9	+S9	−S9	+S9
Recombinant type III collagen		50 μg	100 ± 13	104 ± 7	35 ± 6	37 ± 5	158 ± 45	137 ± 31	280 ± 14	241 ± 12	13 ± 2	11 ± 3
		158 μg	106 ± 6	107 ± 8	31 ± 4	38 ± 4	164 ± 6	142 ± 14	256 ± 7	265 ± 32	11 ± 2	11 ± 1
		500 μg	108 ± 6	111 ± 15	31 ± 6	43 ± 10	163 ± 28	157 ± 2	273 ± 14	245 ± 6	12 ± 2	11 ± 3
		1,582 μg	104 ± 16	99 ± 9	35 ± 8	37 ± 7	168 ± 31	123 ± 25	267 ± 10	285 ± 25	10 ± 1	14 ± 2
		5,000 μg	113 ± 6	101 ± 9	31 ± 3	39 ± 7	139 ± 9	132 ± 25	281 ± 3	263 ± 25	13 ± 3	10 ± 2
Untreated control		—	111 ± 8	105 ± 13	35 ± 5	46 ± 3	155 ± 50	144 ± 3	250 ± 4	268 ± 17	11 ± 2	10 ± 0
Vehicle control	H_2_O	100 μL	105 ± 13	92 ± 5	34 ± 8	47 ± 1	131 ± 18	126 ± 15	253 ± 7	269 ± 5	10 ± 2	12 ± 3
	DMSO	100 μL	106 ± 12	104 ± 7	31 ± 5	33 ± 4	—	170 ± 26	258 ± 11	249 ± 3	—	13 ± 2
Positive control		—	2075 ± 320	1,411 ± 404	1,193 ± 330	1760 ± 406	1,544 ± 269	1,441 ± 368	1,100 ± 286	1,445 ± 38	960 ± 173	995 ± 50

Positive controls and doses: −S9 — TA97a, TA98, TA102: Dacthal (50 μg/per plate); TA100, TA1535: Sodium Azide (1.5 μg/per plate); +S9 — TA97a, TA98, TA100: 2-Aminofluorene (10 μg/per plate); TA102, TA1535: 1,8-Dihydroxyanthraquinone (50 μg/per plate).

**Table 14:** Results of bacterial reverse mutation test with recombinant type III collagen (validation test).

Group		Dose (/per plate)	TA97a		TA98		TA100		TA102		TA1535	
			−S9	+S9	−S9	+S9	−S9	+S9	−S9	+S9	−S9	+S9
Recombinant type III collagen		8 μg	98 ± 12	118 ± 2	31 ± 4	39 ± 8	172 ± 24	118 ± 11	261 ± 10	268 ± 16	12 ± 2	9 ± 2
		40 μg	108 ± 13	104 ± 7	33 ± 8	38 ± 8	146 ± 25	125 ± 24	272 ± 16	284 ± 16	12 ± 2	14 ± 1
		200 μg	92 ± 2	103 ± 8	33 ± 4	36 ± 6	178 ± 17	136 ± 22	251 ± 11	270 ± 20	12 ± 3	12 ± 4
		1,000 μg	100 ± 13	101 ± 5	28 ± 4	43 ± 3	138 ± 30	119 ± 24	263 ± 15	272 ± 14	12 ± 1	12 ± 1
		5,000 μg	103 ± 6	116 ± 5	35 ± 4	33 ± 3	145 ± 19	131 ± 24	270 ± 19	283 ± 14	10 ± 1	11 ± 1
Untreated control		—	111 ± 9	98 ± 12	36 ± 3	39 ± 4	155 ± 12	120 ± 14	279 ± 11	255 ± 13	13 ± 3	13 ± 4
Vehicle control	H_2_O	100 μL	99 ± 15	103 ± 4	38 ± 3	34 ± 3	128 ± 18	116 ± 18	265 ± 21	275 ± 33	12 ± 2	12 ± 4
	DMSO	100 μL	104 ± 14	112 ± 7	39 ± 2	40 ± 2	—	116 ± 14	264 ± 13	271 ± 36	—	12 ± 4
Positive control		—	2087 ± 314	1,176 ± 91	1,378 ± 179	1,684 ± 322	1771 ± 250	1,464 ± 299	1,146 ± 263	955 ± 234	1,113 ± 108	968 ± 94

Positive controls and doses: −S9 — TA97a, TA98, TA102: Dacthal (50 μg/per plate); TA100, TA1535: Sodium Azide (1.5 μg/per plate); +S9 — TA97a, TA98, TA100: 2-Aminofluorene (10 μg/per plate); TA102, TA1535: 1,8-Dihydroxyanthraquinone (50 μg per plate).

**Table 15:** Bacterial reverse mutation test: revertant colonies per plate (main test).

Group		Dose (/per plate)	TA97a		TA98		TA100		TA102		TA1535	
			−S9	+S9	−S9	+S9	−S9	+S9	−S9	+S9	−S9	+S9
Recombinant type III collagen		50 μg	91	112	37	39	189	151	286	254	11	9
			115	102	40	41	179	101	264	235	14	14
			93	98	28	31	106	159	289	233	15	11
		158 μg	110	104	26	35	169	151	264	266	10	10
			109	116	34	38	167	126	252	296	13	11
			99	100	32	42	157	149	252	232	9	11
		500 μg	115	120	38	48	132	155	288	238	13	9
			103	120	28	49	188	158	272	246	10	15
			106	94	26	31	169	157	260	250	14	10
		1,582 μg	88	89	39	38	181	121	278	277	9	16
			105	107	25	40	191	145	265	296	11	16
			119	100	40	33	133	103	258	283	10	9
		5,000 μg	118	102	28	47	131	129	284	285	14	9
			106	109	32	33	137	158	278	236	15	8
			116	92	33	37	148	109	282	267	9	12
Untreated control		—	118	118	32	43	172	142	255	287	10	10
			113	92	40	49	99	147	249	256	10	10
			103	104	32	45	195	143	247	260	14	10
Vehicle control	H_2_O	100 μL	91	91	40	46	147	117	251	266	9	14
			117	88	25	48	135	143	261	266	12	14
			106	97	37	47	111	117	248	274	9	8
	DMSO	100 μL	112	97	36	29	—	141	254	247	—	12
			114	105	27	37	—	191	270	252	—	11
			93	111	30	33	—	179	250	249	—	15
Positive control		—	1828	1,656	1,044	1,436	1736	1,140	968	1,452	856	1,052
			2,436	1,632	964	2,216	1,660	1,332	1,428	1,480	864	960
			1960	944	1,572	1,628	1,236	1852	904	1,404	1,160	972

**Table 16:** Bacterial reverse mutation test: revertant colonies per plate (validation test).

Group		Dose (/per plate)	TA97a		TA98		TA100		TA102		TA1535	
			−S9	+S9	−S9	+S9	−S9	+S9	−S9	+S9	−S9	+S9
Recombinant type III collagen		8 μg	112	115	35	47	158	115	262	252	10	11
			90	119	27	31	158	131	251	284	12	8
			93	119	31	38	200	109	270	267	14	8
		40 μg	117	100	25	31	166	127	263	290	14	14
			94	112	40	46	118	148	263	295	12	15
			114	101	35	36	155	101	290	266	11	14
		200 μg	90	102	29	33	182	113	242	285	9	12
			94	96	33	31	192	138	249	248	13	8
			92	112	37	43	159	156	263	278	15	16
		1,000 μg	89	98	25	48	166	134	276	266	13	10
			114	88	27	39	107	103	267	250	11	14
			96	118	32	43	140	120	246	299	12	11
		5,000 μg	106	116	37	37	164	126	285	279	9	10
			108	111	31	31	145	109	249	298	11	12
			96	120	38	31	127	157	276	271	11	11
Untreated control		—	101	112	32	34	163	130	268	246	14	15
			116	90	37	41	161	104	281	270	15	15
			117	92	38	42	142	127	289	249	9	8
Vehicle control	H_2_O	100 μL	88	108	34	32	139	102	289	288	12	16
			92	101	39	33	107	136	256	300	11	9
			116	100	40	38	138	110	251	237	14	10
	DMSO	100 μL	88	119	37	38	—	128	257	285	—	15
			110	105	38	40	—	101	279	298	—	12
			114	112	41	41	—	118	255	231	—	8
Positive control		—	1804	1,212	1,508	1916	1964	1772	1,192	1,224	1,200	924
			2,424	1,244	1,172	1,316	1860	1,176	864	800	992	1,076
			2032	1,072	1,448	1820	1,488	1,444	1,384	840	1,148	904

##### 3.1.2.2 The mammalian *in vivo* micronucleus test

Experimental results (Tables 17, 18) showed that no abnormal clinical signs or deaths occurred in any group during the dosing period, and initial body weights were balanced across groups (*p* > 0.05). These findings indicate that the test sample did not induce non-specific toxicity within the experimental dose range, eliminating the interference of abnormal animal conditions on subsequent indicators.

**Table 17:** Statistical summary table of animal body weight for micronucleus test in bone marrow polychromatic erythrocytes of mice.

Group	Sex	Initial body weight/g
Vehicle control group	Female	26.6 ± 1.3
	Male	27.5 ± 1.8
Low dose group	Female	26.8 ± 1.8
	Male	27.4 ± 1.6
Mid dose group	Female	26.7 ± 1.8
	Male	27.7 ± 1.7
High dose group	Female	26.6 ± 1.6
	Male	27.5 ± 1.4
Positive control group	Female	26.6 ± 1.4
	Male	27.9 ± 2.1

**Table 18:** Results of micronucleus test in bone marrow polychromatic erythrocytes of mice.

Group	Dose (kg·bw)	Sex	Number of animals (*n*)	Microscopic RBC count (count)	Number of PCEs with micronuclei (count)	Micronucleus frequency (‰, $\bar{x}$ ± s)	PCE/RBC	PCE/RBC ( $\bar{x}$ ± s)
Purified water	20 mL	Female	5	10,000	15	1.5 ± 0.4	470/1000	0.47 ± 0.08
		Male	5	10,000	15	1.5 ± 0.5	408/1000	0.41 ± 0.04
Submitted sample	2,500 mg	Female	5	10,000	17	1.7 ± 0.8	451/1000	0.45 ± 0.07
		Male	5	10,000	16	1.6 ± 0.4	454/1000	0.48 ± 0.10
	5,000 mg	Female	5	10,000	14	1.4 ± 0.4	428/1000	0.43 ± 0.06
		Male	5	10,000	14	1.4 ± 0.4	430/1000	0.43 ± 0.05
	10,000 mg	Female	5	10,000	17	1.7 ± 0.7	443/1000	0.44 ± 0.03
		Male	5	10,000	17	1.7 ± 0.3	402/1000	0.40 ± 0.03
Cyclophosphamide	40 mg	Female	5	10,000	276	27.6 ± 4.2**	454/1000	0.45 ± 0.08
		Male	5	10,000	277	27.7 ± 5.3**	420/1000	0.42 ± 0.04

**Compared with the vehicle control group, *p* < 0.01.

The ratio of polychromatic erythrocytes to total red blood cells (PCE/RBC) is a key indicator for assessing bone marrow hematopoietic function. In this study, the PCE/RBC ratios of the test sample’s dose groups and the positive control group were all no lower than 20% of those in the vehicle control group (females ≥ 94/1000; males ≥ 81.6/1000), with no statistically significant differences between groups (*p* > 0.05). This suggests that the test sample did not inhibit the proliferation or differentiation of bone marrow hematopoietic stem cells, maintaining a normal bone marrow microenvironment and ensuring the representativeness of samples for micronucleus detection.

Micronucleus frequency is a core indicator reflecting chromosomal damage or spindle apparatus dysfunction. Results revealed that the micronucleus frequencies in female and male mice from the test sample’s dose groups (females: 1.4–1.7‰; males: 1.4–1.7‰) were not significantly different from those in the vehicle control group (females: 1.5 ± 0.4‰; males: 1.5 ± 0.5‰) (*p* > 0.05). Furthermore, the micronucleus frequencies in all dose groups fell within the historical range of the negative control group (0.90–3.50‰). These findings indicate that the test sample did not induce chromosomal breaks or spindle apparatus dysfunction, showing no potential genotoxic risk. Notably, the positive control group (cyclophosphamide at 40 mg/kg·bw) exhibited a significantly elevated micronucleus frequency (females: 27.6 ± 4.2‰; males: 27.7 ± 5.3‰; *p* < 0.01), which aligned with the laboratory’s historical positive control range (15.90–44.60‰). This validated the sensitivity of the experimental system to known genotoxic substances and indirectly ruled out experimental procedural errors, enhancing the credibility of the negative conclusion.

Additionally, the high dose (10,000 mg/kg·bw) was set based on the rat acute oral LD_50_ > 10,000 mg/kg·bw, with intermediate and low doses decreasing by a 2-fold spacing, covering potential toxicity thresholds. The absence of a dose–response relationship across groups further supports the conclusion of “no genotoxicity”. Although the micronucleus test does not cover other genotoxic endpoints such as gene mutations or abnormal DNA damage repair, comprehensive analysis of animal conditions, body weights, PCE ratios, and micronucleus frequencies indicates that, under the experimental conditions, rhCol III did not affect the micronucleus frequency of mouse bone marrow PCEs and did not exhibit potential genotoxic risks related to chromosomal damage or spindle apparatus dysfunction. This provides critical experimental evidence for its safety evaluation.

##### 3.1.2.3 *In vitro* mammalian cell chromosome aberration assay

Table 19 presents the statistical analysis results of chromosomal aberrations in the Chinese Hamster Lung (CHL) cell line. The chromosomal aberration rates of CHL cells in the positive control groups (MMS and CP) were 12.0 and 10.0%, respectively. Compared with the corresponding solvent control group, the differences were statistically significant (*p* < 0.01). For each dose group of rhCol III, there were no statistically significant differences in the chromosomal aberration rates of CHL cells when compared with the corresponding solvent control group (*p* > 0.05).

**Table 19:** Effect of recombinant type III collagen on chromosome aberration rate in CHL cells (4 h).

Group		Dose (/mL)	S9	Metaphase phase cells	Numerical aberrations			Structural aberrations								Aberrant chromosomes number	Cell aberration number	Cell aberration rate (%)
					aneu	poly	ed	brk	mc	cr	ar	ci	dmc	gaps	nsc			
Solvent control		−	−	100	0	0	0	1	0	0	0	0	0	0	0	1	1	1.0
			+	100	1	0	0	0	0	0	0	0	0	0	1	2	2	2.0
Recombinant type III collagen		1,250 μg	−	100	0	0	0	1	0	0	0	0	0	0	0	1	1	1.0
			+	100	0	0	0	0	0	0	0	0	0	0	0	0	0	0
		2,500 μg	−	100	0	0	0	1	0	0	0	1	0	0	0	2	2	2.0
			+	100	0	0	0	0	0	0	0	0	0	0	1	1	1	1.0
		5,000 μg	−	100	0	0	0	0	1	0	0	0	0	0	0	1	1	1.0
			+	100	0	0	0	1	0	0	0	0	0	0	0	1	1	1.0
Positive control	MMS	20 μg	−	100	0	0	0	12	1	0	0	1	0	2	3	17	12	12.0**
	CP	10 μg	+	100	0	0	0	9	0	0	0	1	0	1	1	11	10	10.0**

Aneu, aneuploidy; poly, polyploidy; ed, endoreduplication; brk, breaks; mc, minichromosomes; cr, centromeric rings; ar, acentric rings; ci, chromatid interchange; dmc, double minichromosomes; gap, gaps; nsc, non-specific changes. Non-specific chromosomal structural changes in this table include pulverization, centromere elongation, adhesion (stickiness), and dicentric or polycentric chromosomes; gaps are not included in the number of aberrant cells. **Compared with the solvent control group, the difference was statistically significant, *p* < 0.01.

Since the test substance did not show obvious chromosomal aberration-inducing results under the 4-h exposure time, an additional verification experiment was conducted. Under the same test substance concentration conditions, the exposure time of the test substance to the cells was extended to 24 h. The results in Table 20 show that, for each dose group of rhCol III, there were no statistically significant differences in the chromosomal aberration rates of CHL cells when compared with the corresponding solvent control group. No dose–response relationship was observed between different dose groups. These results indicate that under the conditions of this experiment, rhCol III did not induce chromosomal aberrations in CHL cells.

**Table 20:** Effect of recombinant type III collagen on chromosome aberration rate in CHL cells (24 h).

Group	Dose (/mL)	S9	Metaphase phase cells	Numerical aberrations			Structural aberrations								Aberrant chromosomes number	Cell aberration number	Cell aberration rate (%)
				aneu	poly	ed	brk	mc	cr	ar	ci	dmc	gaps	nsc			
Solvent control	—	—	100	0	0	0	1	0	0	0	0	0	0	0	1	1	1.0
Recombinant type III collagen	1,250 μg	—	100	0	0	0	1	0	0	0	0	0	0	0	1	1	1.0
	2,500 μg		100	0	0	0	1	0	0	0	0	0	1	0	1	1	1.0
	5,000 μg		100	0	0	0	3	0	0	0	0	0	0	0	3	2	2.0
Positive control MMS	10 μg	—	100	0	0	0	10	0	0	0	1	0	1	1	12	11	11.0**

Aneu, aneuploidy; poly, polyploidy; ed, endoreduplication; brk, breaks; mc, minichromosomes; cr, centromeric rings; ar, acentric rings; ci, chromatid interchange; dmc, double minichromosomes; gap, gaps; nsc, non-specific changes. Non-specific chromosomal structural changes in this table include pulverization, centromere elongation, adhesion (stickiness), and dicentric or polycentric chromosomes; gaps are not included in the number of aberrant cells. **Compared with the solvent control group, the difference was statistically significant, *p* < 0.01.

### 3.2 Potential oral beauty efficacy

#### 3.2.1 Anti-aging action

**Figure 4:**
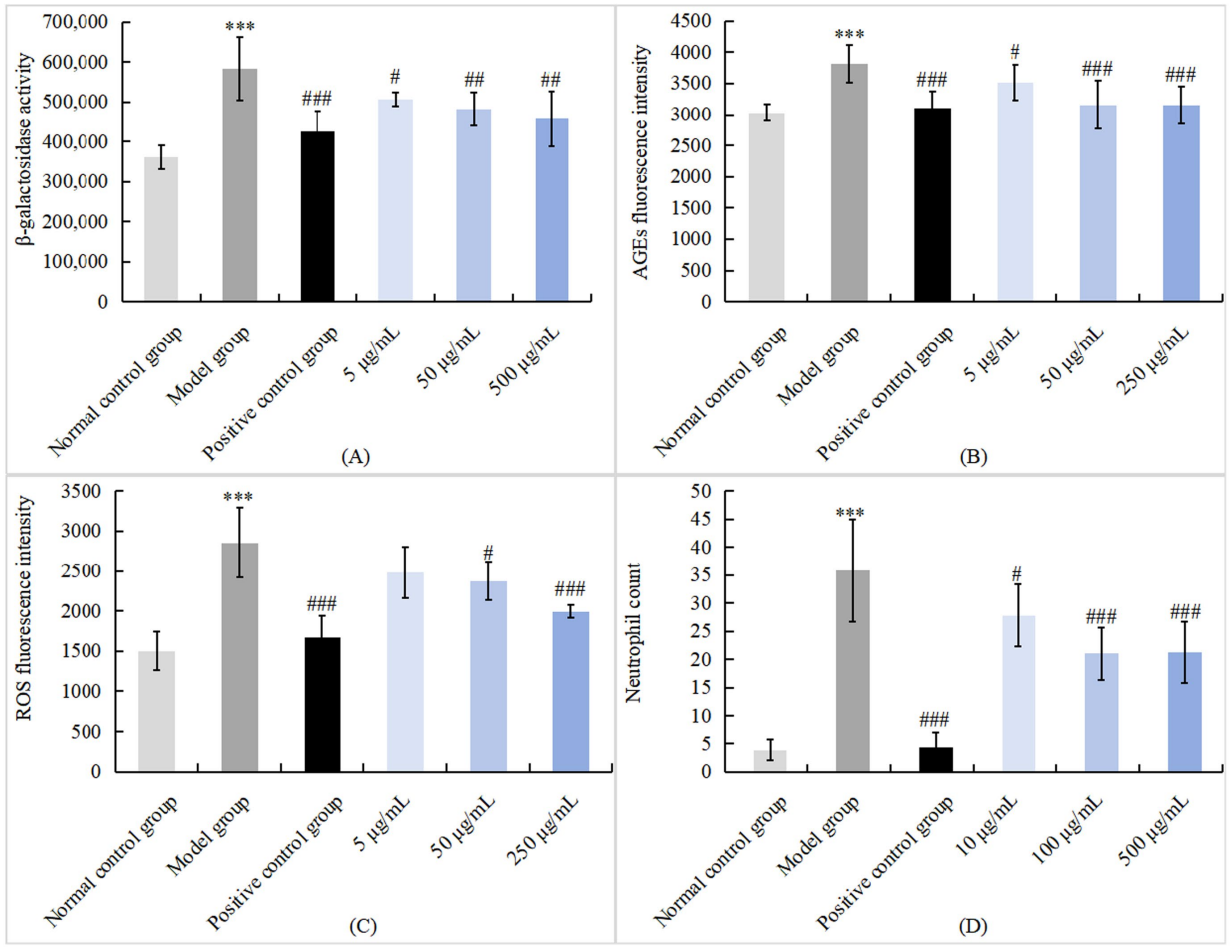
Potential oral beauty efficacy data column graph combination. Compared with the normal control group, ***p < 0.001; compared with the model group, #*p* < 0.05, ##*p* < 0.01, ###*p* < 0.001. **(A)** Anti-aging action, bar chart of blue staining intensity of *β*-galactosidase; **(B)** anti-glycation action, AGEs fluorescence intensity bar chart; **(C)** anti-oxidation action, bar chart of ROS fluorescence signal intensity; **(D)** anti-inflammatory action, neutrophil count bar chart (chart data are provided in Appendix D).

**Figure 5:**
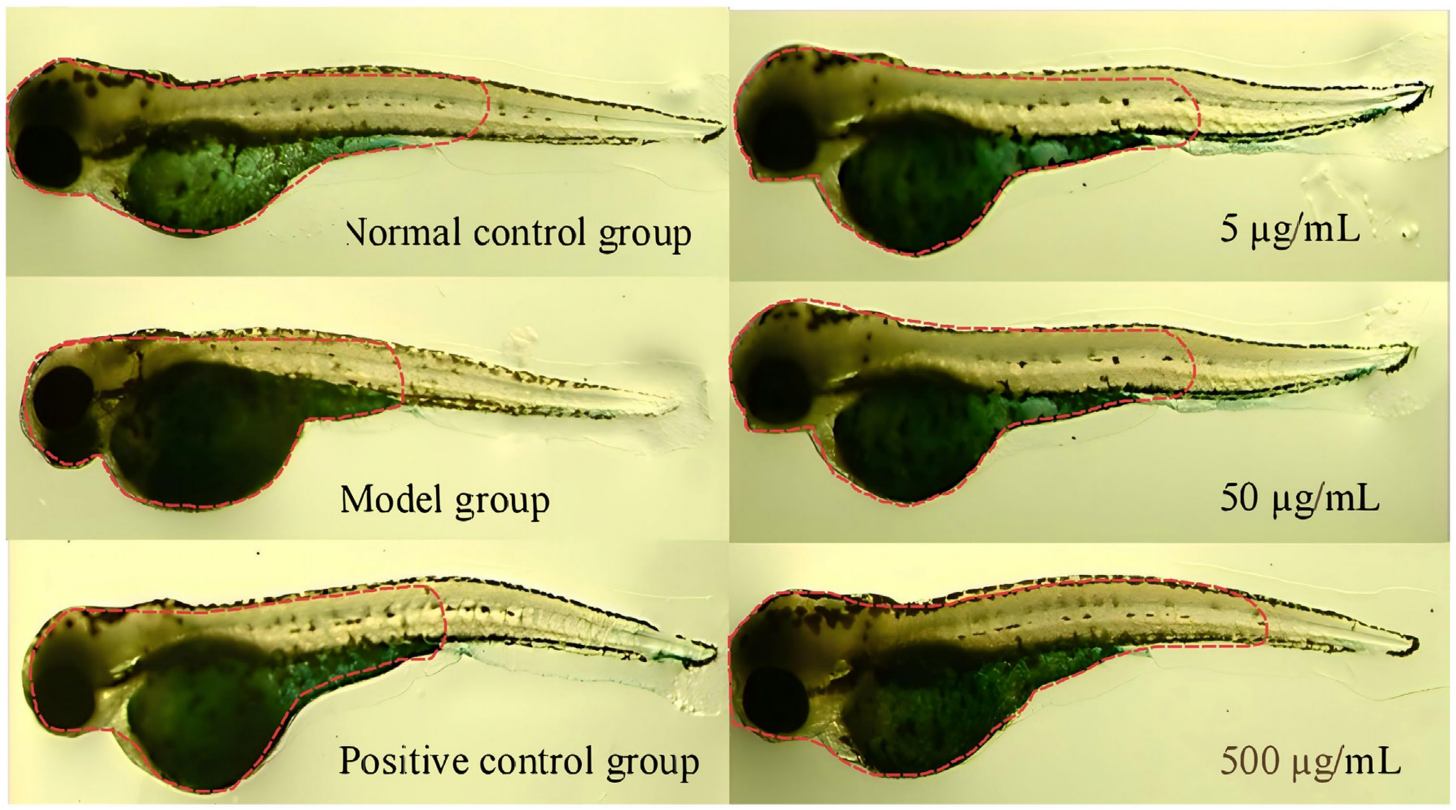
β-Galactosidase staining diagram of zebrafish in each experimental group. Phenotype of blue staining intensity: the X-Gal substrate is catalyzed by senescence-specific β-galactosidase to generate a deep blue product. Under an optical microscope, cells or tissues expressing β-galactosidase that have turned deep blue can be observed. In zebrafish larvae, the staining changes were particularly prominent in regions such as the abdominal yolk sac and the head.

The blue staining intensity of *β*-galactosidase in zebrafish treated with rhCol III is shown in Figure 4A. As seen from Figure 5, under microscopic observation, the model group exhibited deeper blue staining with a large area of blue staining in the abdomen. In contrast, the blue staining in the sample groups at all concentrations was lighter than that in the model group to varying degrees.

The statistical results of the quantitative analysis in Table 7 showed that the β-galactosidase blue staining intensity of zebrafish in the model group treated with standard dilution water for 5 days was 583035.60. After 5 days of treatment with rhCol III, the β-galactosidase blue staining intensities in zebrafish of the low-concentration group, intermediate-concentration group, and high-concentration group were 507152.20, 481017.30, and 457270.90, respectively. The β-galactosidase activities in the three sample groups (low, intermediate, and high concentrations) decreased sequentially, with reductions of 13.02% (*p* < 0.05), 17.50% (*p* < 0.01), and 21.57% (*p* < 0.01) compared with the model group, respectively. These results indicate that rhCol III has a significant inhibitory effect on the β-galactosidase activity in zebrafish.

#### 3.2.2 Anti-glycation action

Compared with the normal control group, the fluorescence intensity of AGEs in the model group was significantly increased (*p* < 0.05); compared with the model group, the fluorescence intensity of AGEs in the positive control group was significantly decreased (~ 18.75%, *p* < 0.05), indicating that the experimental system was effective.

The effects of rhCol III on the fluorescence intensity of AGEs in zebrafish are shown in Figure 4B. After 3 days of treatment with standard dilution water, the fluorescence intensity of AGEs in the model group was 3821.20. After 3 days of treatment with rhCol III, the fluorescence intensities of AGEs in zebrafish of the low-concentration group, intermediate-concentration group, and high-concentration group were 3513.00, 3155.00, and 3145.90, respectively. Compared with the model group, these values decreased by 8.07% (*p* < 0.05), 17.43% (*p* < 0.001), and 17.67% (*p* < 0.001), respectively. These results indicate that rhCol III has a significant reducing effect on the fluorescence intensity of AGEs in zebrafish.

#### 3.2.3 Anti-oxidation action

Compared with the normal control group, the ROS fluorescence intensity in the model group was significantly increased (*p* < 0.05); compared with the model group, the ROS fluorescence intensity in the positive control group was significantly decreased (~ 41.64%, *p* < 0.05), indicating that the experimental system was effective.

The effects of rhCol III on the ROS fluorescence intensity of zebrafish are shown in Figure 4C. After treatment with standard dilution water for 24 h, the ROS fluorescence intensities of the high, intermediate, and low dose model groups were reduced by 13.03, 16.93% (*p* < 0.05), and 30.22% (*p* < 0.001) respectively compared with the model group. These results indicate that rhCol III has a significant inhibitory effect on the ROS fluorescence intensity of zebrafish.

#### 3.2.4 Anti-inflammatory action

**Figure 6:**
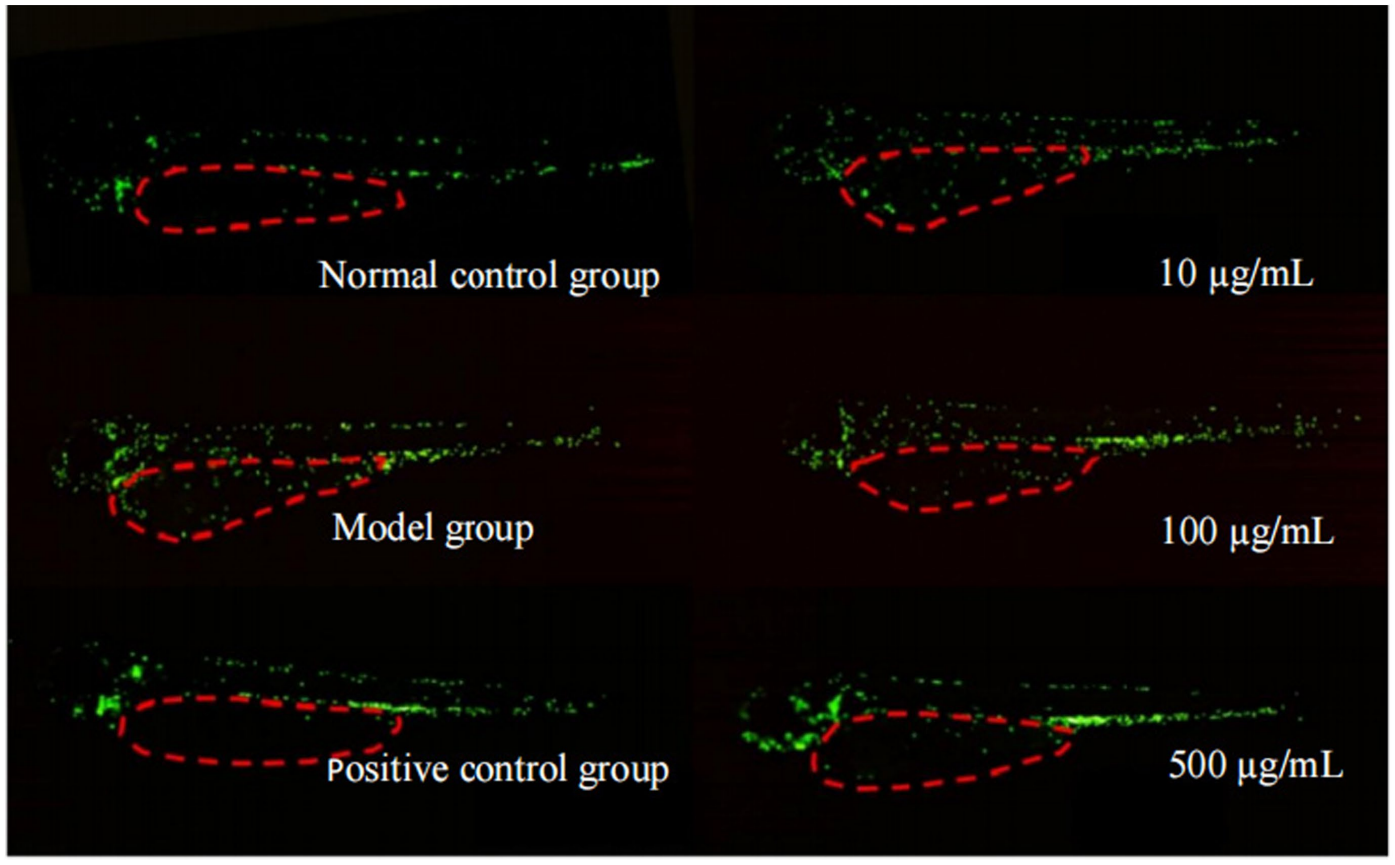
Fluorescence phenotyping of neutrophils. Capture fluorescence images under a microscope and count the neutrophils. In the experiment, transgenic neutrophil fluorescent fish (appearing green) were used. Under the fluorescent microscope, it was observed that the number of neutrophils in the yolk sac of zebrafish was significantly higher than that of normal zebrafish.

Fluorescence phenotyping of neutrophils is demonstrated in Figure 6. Compared with the normal control group, the neutrophil count in the model group was significantly increased (*p* < 0.05); compared with the model group, the neutrophil count in the positive control group was significantly decreased (*p* < 0.05), indicating that the experimental system was effective.

As shown in Figure 4D, after treating zebrafish with rhCol III, the neutrophil counts were reduced by 22.56% (*p* < 0.05), 41.50% (*p* < 0.001), and 40.67% (*p* < 0.001) compared with the model group. These results indicate that rhCol III has a significant inhibitory effect on neutrophils in zebrafish. Under the conditions of this experiment, rhCol III exhibits the efficacy of improving sensitive skin (anti-inflammatory effect).

## 4 Discussion

The findings of this study preliminarily confirmed that recombinant humanized type III collagen (rhCol III) is safe for oral consumption and oral anti-aging efficacy. The study demonstrated that rhCol III exhibited favorable biosafety under the experimental conditions employed. Subchronic toxicity tests revealed that this protein did not exert any adverse effects on the growth, development, physiological metabolism, or major organs of rats. Meanwhile, a series of genotoxicity tests yielded negative results, supporting that it poses no risks of genotoxicity. The chromosome aberration assay included in this study is primarily intended to evaluate structural chromosomal aberrations, assessing the potential genotoxicity of the test substance by determining its ability to induce structural damage. While an increase in polyploidy may indicate the possibility of numerical chromosomal aberrations, the present method is not sufficiently accurate for detecting such numerical abnormalities. However, overall, most mutagens induce chromatid-type aberrations, with chromosomal-type aberrations occurring only occasionally. Therefore, the conclusions drawn from our experiment are sufficiently reliable for reference. In summary, consistent findings from the systematic evaluation encompassing subchronic toxicity to genotoxicity indicated that rhCol III is safe within the tested dose range, providing crucial scientific evidence for its further development and application.

Skin aging results from the combined effects of intrinsic factors and extrinsic factors (e.g., photoexposure, oxidative stress, and glycation). Oral rhCol III has been shown to mitigate skin aging, and its mechanism can be systematically interpreted from two perspectives: bioavailability and multi-pathway synergy.

From a bioavailability perspective, the oral efficacy of rhCol III is unlikely to be mediated directly by the intact triple-helical structure; rather, it is more plausibly driven by bioactive peptides generated during gastrointestinal digestion. Studies have shown that after ingestion of collagen hydrolysates, specific peptide fragments can be detected in plasma, and certain metabolites can be transported via the circulation to skin tissue, providing a biological basis for systemic effects of collagen peptides (26).

At the molecular level, collagen peptides may exert anti–skin-aging effects through several interconnected pathways. First, with respect to extracellular matrix metabolism, collagen peptides can serve as biosynthetic substrates to support collagen production and can promote collagen and hyaluronic acid synthesis by modulating cytokine signaling; meanwhile, they may reduce collagen degradation by decreasing the activity of collagenolytic enzymes such as matrix metalloproteinase-3 (MMP-3). Second, regarding antioxidative and anti-inflammatory actions, collagen peptides can reduce intracellular reactive oxygen species (ROS) and thereby alleviate oxidative injury (27–29). Evidence suggests that these effects may involve activation of the endogenous Nrf2/ARE defense pathway, which enhances antioxidant capacity and can downregulate inflammation through suppression of NF-κB signaling. In zebrafish and cellular models, collagen peptides have been reported to inhibit ROS accumulation and MAPK/NF-κB signaling, leading to reduced expression of inflammatory mediators and diminished inflammatory cell recruitment, consistent with a coupled anti-oxidative–anti-inflammatory mechanism (30, 31). In addition, collagen peptides also exhibit anti-glycation activity. They may inhibit the formation of advanced glycation end products (AGEs) and attenuate downstream AGE–RAGE signaling. Notably, AGE precursors (e.g., methylglyoxal) are generated more rapidly under oxidative conditions, whereas AGE–RAGE interactions can further activate ROS production and NF-κB–mediated inflammatory pathways, forming a positive feedback loop of “glycation–oxidation–inflammation” (32–34). Therefore, by reducing oxidative stress and inflammatory signaling, collagen peptides may help interrupt this loop and decrease AGE accumulation.

To investigate whether rhCol III has oral anti-aging efficacy, we established a zebrafish skin model using D-galactose. D-galactose is an aldohexose and a reducing sugar naturally present in the human body, as well as in numerous foods such as beets, plums, cherries, figs, celery, and certain dairy products (35). D-galactose can induce skin aging by disrupting the structure of collagen in the dermis (36) and significantly reducing the level of hydroxyproline in the skin (37). Experiments on animal tissues have shown that the content of collagen fibers and elastin in the skin tissues of animals treated with D-galactose is significantly decreased (37–39). The use of D-galactose in aging models has been demonstrated to increase the accumulation of AGEs (40).

The elevation of *β*-galactosidase activity in senescent cells is considered to be one of the markers of skin aging (41). Dermal staining of senescence-associated β-galactosidase (SA-β-galactosidase) in the skin also exhibits a significant age-related correlation (42). In the aging zebrafish model established by D-galactose, it was observed that the activity of β-galactosidase in zebrafish decreased following the intake of rhCol III, with the activity reduction exceeding 10% across all three tested concentrations compared to the model group, and this result was more similar to that of the positive control group treated with vitamin E. This indicates that rhCol III exerts anti-aging activity in zebrafish.

To further explore mechanisms underlying these effects, we assessed anti-glycation, antioxidant, and anti-inflammatory activities using zebrafish models.

Glycation is one of the causes of aging in human skin. Glycation, as defined in the non-enzymatic glycation aging theory, refers to the accumulation of AGEs (43). AGEs are formed through the non-enzymatic condensation of the carbonyl group of reducing sugars with the free amino groups of nucleic acids, proteins, or lipids, followed by further rearrangement to generate stable and irreversible end-products (44). The binding of AGEs to proteins also impairs the structure and function of proteins in the dermis, thereby accelerating skin aging (45). Additionally, in photoaged skin models, the formation and accumulation of AGEs influence the expression of various pro-inflammatory cytokines (46).

Recombinant humanized type III collagen (rhCol III) may have the ability to alleviate skin aging caused by AGE accumulation through anti-glycation effects. In order to verify this speculation, leveraging the fluorescent properties of AGEs, we induced AGE formation in zebrafish under high-glucose conditions, followed by the administration of rhCol III. The anti-glycation efficacy was then evaluated by quantifying the inhibitory effect on AGE levels using fluorescence-based quantification. The experimental results showed that after continuous intake of rhCol III for three days, the fluorescence intensity of AGEs in zebrafish decreased by approximately 10–20% compared to the control group, and this result was more similar to that of the positive control group treated with carnosine. This is consistent with that rhCol III can reduce the elevation of *in vivo* AGE levels induced by high-concentration glucose. Furthermore, it can be preliminarily concluded that within a certain concentration range, the in vivo anti-glycation capacity of rhCol III increases with the increase in intake.

AGEs, along with certain mechanical damages, can induce the oxidative stress and inflammatory responses by activating the receptor for advanced glycation end-products (RAGE) on the cell surface (47). The redox balance of the organism is a crucial indicator for evaluating the health and youthfulness of the body. The redox theory of aging suggests that with increasing age, the flexibility of the human body’s redox network, which forms during interactions with the external environment, continuously declines. The redox metabolome and redox proteome play key roles in maintaining the adaptability of the human body to the environment (48), and the decreased adaptability caused by this irreversible characteristic leads to human aging. Excessive oxidation can also contribute to other hallmarks of aging to a certain extent, such as the accumulation of macromolecular aggregates, disruption of protein homeostasis, impairment of intercellular communication, and damage to the protective barrier function (49).

ROS are chemically reactive oxygen-containing substances, including peroxides, superoxides, hydroxyl radicals, and others. The intracellular ROS are natural by-products of the normal oxygen metabolism in organelles such as mitochondria (50), and they can affect cellular signal transduction and in vivo redox balance. Hydrogen peroxide can generate oxygen free radicals, and when this oxidative stress exceeds the body’s capacity for recovery, an oxidative stress response occurs. Glutathione is a natural antioxidant that can reduce the accumulation of ROS in cells and has potential efficacy in combating melanin production and chloasma (51). The ROS indicator 2’,7’-Dichlorodihydrofluorescein diacetate (DCFH-DA) can produce relatively stable and high-intensity fluorescence under the action of intracellular ROS. After adding it to the culture system of zebrafish larvae for a certain period, the production of ROS in zebrafish can be assessed by detecting the fluorescence intensity.

The results of the zebrafish antioxidant test showed that after the intake of rhCol III, the fluorescence signal intensity of ROS in hydrogen peroxide-treated zebrafish was significantly reduced compared to that in model group (before collagen intake). Moreover, this reduction effect became more pronounced with the increase in the intake concentration of collagen. When the concentration reached 250 μg/mL, the fluorescence intensity decreased by more than 30% compared to the model group, and the result was more similar to that of the positive control group treated with glutathione. These results indicate that the intake of rhCol III can partially counteract the oxidative damage caused by ROS in zebrafish, further demonstrating that rhCol III possesses antioxidant efficacy.

The anti-inflammatory process may also help regulate signaling pathways related to oxidative stress. To corroborate the previous experimental results and investigate whether rhCol III also exhibits anti-inflammatory efficacy, we conducted a skin inflammation modeling experiment using SDS and employed transgenic zebrafish with fluorescent neutrophils (exhibiting green fluorescence) to observe the migration and aggregation quantity of neutrophils under a fluorescence microscope. SDS is an anionic surfactant commonly used as an emulsifier and cleanser in drug carriers and cosmetics (52). It can disrupt the cellular barrier function and induce the appearance of numerous vesicles and lipid accumulation in the cytoplasm of keratinocytes, leading to parakeratosis to a certain extent (53). Damaged keratinocytes then release inflammatory factors, thereby triggering skin inflammation. During this process, immune cells migrate and aggregate to the site of epidermal inflammation under the action of chemokines, among which neutrophils exhibit the highest sensitivity and specificity.

The experimental results showed that after treating zebrafish with rhCol III, the number of neutrophils was significantly reduced compared to the model group. Specifically, the number decreased by approximately 20% in the low-dose group and by up to 40% in the intermediate- and high-dose groups. These results were more similar to that of the positive control group treated with indomethacin and indicate that the intake of rhCol III exerts a favorable anti-inflammatory effect in the zebrafish model.

In addition to the anti-glycation, antioxidant, and anti-inflammatory actions discussed above, maintenance of epidermal barrier homeostasis is a key dimension of anti-aging. Evidence from oral collagen peptide studies support that barrier improvement can be mediated through enhancement of stratum corneum water-holding components and keratinocyte differentiation programs. Clinical trials reported that oral collagen peptides increased stratum corneum/epidermal water content, reduced transepidermal water loss (TEWL), and elevated natural moisturizing factor (NMF) levels, suggesting strengthened “inside-out” barrier function. Likewise, 12-week supplementation with enzymatically decomposed collagen peptides increased both NMF and total ceramides (including ceramide subclasses) in the stratum corneum, supporting reinforcement of the lipid–protein barrier (54, 55). Mechanistically, in a UVB-challenged model, dietary collagen peptides upregulated NMF-related barrier proteins (filaggrin and involucrin) and promoted hyaluronic-acid homeostasis by increasing HAS1/HAS2 while downregulating HYAL1/HYAL2, thereby alleviating barrier disruption and dehydration (56). Furthermore, tight-junction integrity is closely linked to inflammatory amplification; decreased claudin-1 impairs epidermal barrier function and can induce keratinocyte-autonomous IL-1β expression (57). Collectively, these findings provide a mechanistic rationale that rhCol III may contribute to oral anti-aging benefits partly by supporting epidermal barrier integrity (NMF/ceramide/HA pathways and junctional stability), in parallel with its observed antioxidant and anti-inflammatory activities.

Compared with conventional animal-derived collagens (e.g., bovine/porcine) that are typically extracted from tissues, rhCol III offers a sequence-defined ingredient with improved batch-to-batch consistency and traceability, while reducing dependence on animal raw materials and associated variability in source control. From a food-safety governance perspective, ruminant-derived collagen/gelatin is subject to dedicated TSE/BSE risk management, and EFSA has recently performed a quantitative risk assessment specifically for collagen/gelatin produced from ruminant bones, underscoring the importance of controlled sourcing and processing when animal tissues beyond hides/skins are involved (58). Marine collagens may alleviate certain ruminant-related concerns; however, IgE sensitization to fish gelatin/collagen has been documented in subsets of fish-allergic individuals, indicating that allergenicity remains a population-specific consideration for some animal-derived collagen products (59, 60). In contrast, recombinant/precision-fermentation approaches enable tighter control over amino-acid sequence and manufacturing conditions, which is advantageous for product standardization; nevertheless, product-specific safety evaluation should still address impurities related to the production host and process-derived specifications (61, 62). Importantly, recombinant collagen ingredients have entered the food regulatory pathway: the U.S. FDA issued a “no questions” response to a GRAS notice for a collagen polypeptide produced by engineered *Escherichia coli* K-12, illustrating the feasibility of regulatory compliance for fermentation-derived collagen ingredients (63).

Although the zebrafish assays provide valuable first-pass evidence supporting the anti–skin-aging potential of orally administered rhCol III, several limitations should be considered when extrapolating these findings to long-term human use. First, zebrafish larvae differ from humans in metabolic capacity and immune maturation. During early development, larvae predominantly rely on innate immunity, whereas the adaptive immune system requires substantially longer to become morphologically and functionally mature; therefore, inflammatory readouts may not fully recapitulate the complex human immune milieu (64). Second, the immersion exposure used in this study cannot faithfully model the gastrointestinal “digestion–absorption–first-pass metabolism” process associated with oral intake. In larval zebrafish, immersion delivery also makes it difficult to precisely control the route and timing of intestinal exposure, and the amount ingested can vary across individuals, which may introduce variability in the effective internal dose (65, 66). This is particularly relevant for collagen-based ingredients because dietary collagen is expected to be digested into smaller peptides before absorption. Consistent with this rationale, collagen peptides have been detected in human blood after ingestion of collagen/gelatin hydrolysates, and a high-tripeptide collagen hydrolysate has been shown to deliver into the bloodstream and skin, supporting a peptide-mediated mechanism while underscoring the importance of gastrointestinal processing for systemic exposure (26, 67). Third, several efficacy endpoints in this work relied on fluorescence readouts (e.g., DCFH-DA–based ROS signals and fluorescence-associated AGE signals), which represent surrogate phenotypes and do not resolve specific ROS species or chemically defined AGE adducts (such as CML and pentosidine) and their downstream signaling pathways (33). Notably, the low-dose rhCol III group (5 μg/mL) showed a trend toward reduced ROS fluorescence but did not reach statistical significance (*p* = 0.0554), which may reflect a smaller effect size at low exposure, limited statistical power, and the known methodological limitations and sensitivity of commonly used fluorescent probes (68, 69). Future studies should increase statistical power and incorporate more specific oxidative stress endpoints (e.g., antioxidant enzyme activities, lipid peroxidation products) and pathway markers to strengthen causal inference.

In summary, rhCol III demonstrated favorable safety profiles and preliminarily exhibited potential oral anti-aging effects mediated through anti-glycation, antioxidant, and anti-inflammatory mechanisms. Future studies may integrate transcriptomic analyses and other advanced methodologies to further elucidate the specific regulatory mechanisms underlying oxidative stress and inflammatory signaling pathways (70), thereby strengthening the theoretical foundation for its application.

## Data Availability

The raw data supporting the conclusions of this article will be made available by the authors, without undue reservation.
