# Supplementary material for: Evaluation of the safety and functional effects of recombinant humanized type III collagen in food toxicology
Source: Front Med (Lausanne). 2026 Feb 12;13:1765276. doi: 10.3389/fmed.2026.1765276 (PMC12936025; doi:10.3389/fmed.2026.1765276)
Supplement: Supplementary file 1 [file Data_Sheet_1.pdf]

Appendix A

Effects of Recombinant Humanized Type III Collagen on Body Weight, Food Consumption, and Food Utilization in Rats

Table 1 Summary of Interim Satellite Group Body Weights of Rats in the 90-day Oral Toxicity Test (g, $\overline{x}$ ± s)

Sex	Weighing time	Dose group (n=5)	
		Interim control satellite group	Interim high-dose satellite group
Female	Initial body weight	77.2 ± 6.4	75.8 ± 3.6
	1	135.1 ± 9.3	135.1 ± 7.7
	2	184.6 ± 11.6	183.6 ± 8.2
	3	211.1 ± 16.8	212.2 ± 11.9
	4	236.2 ± 17.2	232.9 ± 16.7
	5	255.0 ± 25.4	254.6 ± 18.0
	6	276.9 ± 25.5	280.5 ± 19.8
	Total weight gain	199.7 ± 19.9	204.7 ± 17.0
Male	Initial body weight	79.6 ± 6.0	79.9 ± 4.9
	1	148.9 ± 11.6	146.3 ± 8.0
	2	220.3 ± 18.6	205.9 ± 5.6
	3	290.0 ± 19.7	284.0 ± 9.2
	4	360.7 ± 22.6	344.4 ± 10.0
	5	408.0 ± 29.3	402.0 ± 18.9
	6	464.3 ± 27.6	451.9 ± 31.7
	Total weight gain	384.7 ± 23 9	372.0 ± 28:7

Table 2 Summary of Interim Satellite Group Food Consumption of Rats in the 90-day Oral Toxicity Test (g, $\overline{x}$ ± s)

Sex	Time (week)	Dose group (n=5)	
		Interim control satellite group	Interim high-dose satellite group
Female	1	130.6 ± 8.6	140.3 ± 4.2
	2	158.8 ± 6.6	165.7 ± 7.3

	3	155.1 ± 9.7	162.1 ± 18.6
	4	170.7 ± 9.8	172.7 ± 10.2
	5	161.4 ± 11.2	168.1 ± 13.6
	6	197.2 ± 17.2	203.4 ± 16.3
	Total food consumption (g)	973.8 ± 54.1	1012.4 ± 61.0
Male	1	150.2 ± 8.1	157.1 ± 10.1
	2	190.1 ± 14.5	189.6 ± 5.3
	3	234.4 ± 16.2	280.5 ± 16.1**
	4	261.2 ± 13.8	263.5 ± 5.5
	5	234.1 ± 17.3	252.5 ± 6.7
	6	321.0 ± 26.5	318.4 ± 11.2
	Total food consumption (g)	1391.0 ± 83.7	1461.5 ± 25.9

Note: ** indicates a statistically significant difference compared with the control group ($P < 0.01$).

Table 3 Summary of Interim Satellite Group Food Utilization of Rats in the 90-day Oral Toxicity Test (g, $\overline{x} \pm s$)

Sex	Time (week)	Dose group (n=5)	
		Interim control satellite group	Interim high-dose satellite group
Female	1	44.4 ± 3.0	42.3 ± 2.8
	2	31.2 ± 3.2	29.4 ± 4.5
	3	17.1 ± 4.8	17.8 ± 4.3
	4	14.7 ± 2.7	11.9 ± 3.0
	5	11.4 ± 5.0	13.1 ± 3.2
	6	11.0 ± 4.6	12.7 ± 3.2
	Total food utilization	20.5 ± 1.0	20.2 ± 1.4
Male	1	46.1 ± 2.8	42.4 ± 3.4
	2	37.7 ± 4.2	31.3 ± 5.5
	3	29.8 ± 2.2	28.1 ± 5.4
	4	27.1 ± 2.3	22.9 ± 2.6*
	5	20.2 ± 4.9	22.8 ± 4.7

6	17.7 ± 2.8	15.6 ± 3.7
Total food utilization	27.7 ± 1.8	25.4 ± 1.7

Note: * indicates that the difference was statistically significant compared with the control group (P<0.05).

Table 4 Statistical Summary Table of Animal Body Weight in Each Dose Group of the 90-Day Oral Toxicity Study in Rats (g, $\overline{x}$ ± s)

Sex	Weighing time	Dose Groups (n=10)			
		Control Group	Low Dose Group	Intermediate-dose Group	High Dose Group
Female	Initial body weight	75.4 ± 4.4	76.5 ± 3.6	76 ± 4.7	77.2 ± 4.7
	Week 1	129.9 ± 8.0	138.6 ± 6.0*	130.4 ± 6.7	136 ± 8.3
	Week 2	177.4 ± 17.1	184.6 ± 10.7	178.8 ± 11.2	181.7 ± 15.8
	Week 3	208.4 ± 21.4	218.6 ± 14.7	210.1 ± 12.4	207.6 ± 21.3
	Week 4	231.9 ± 22.4	245.7 ± 13.8	237.8 ± 14.8	231.3 ± 25.9
	Week 5	251.7 ± 26.9	266.4 ± 20.8	253.8 ± 15.9	250.4 ± 30.5
	Week 6	264.7 ± 26.2	282.6 ± 22.6	270.5 ± 17.7	266.8 ± 29.5
	Week 7	274.2 ± 28.1	297.2 ± 26.0	286.6 ± 20.1	274.4 ± 29.8
	Week 8	282 ± 30.3	305.8 ± 24.2	293.1 ± 21.7	285.7 ± 34.5
	Week 9	295.9 ± 32.6	319.6 ± 26.3	305 ± 22.6	298.2 ± 35.1
	Week 10	302.9 ± 32.3	324.1 ± 24.9	308.7 ± 23.6	305 ± 36.5
	Week 11	312.5 ± 36.0	328.7 ± 25.2	314.1 ± 26.7	311.8 ± 40.2
	Week 12	316 ± 38.5	332.5 ± 27.1	317.8 ± 26.1	320.3 ± 37.9
	End of the treatment period	316.3 ± 38.0	334.4 ± 27.4	321.6 ± 29.3	318.5 ± 39.7
	Total weight gain	240.9 ± 36.4	257.9 ± 28.9	245.6 ± 28.1	241.3 ± 40.0
Male	Initial body weight	79.8 ± 4.4	80.2 ± 5.6	80.1 ± 5.5	80.7 ± 4.4
	Week 1	149.2 ± 6.6	146.3 ± 10.9	147.2 ± 8.0	143.7 ± 9.7
	Week 2	217.1 ± 9.2	214.9 ± 17.6	217.8 ± 13.6	212.8 ± 9.4
	Week 3	286.2 ± 13.9	281.0 ± 20.6	294.4 ± 20.5	279.1 ± 14.6
	Week 4	347.3 ± 18.9	344.3 ± 28.3	356.3 ± 26.3	342.6 ± 18.0
	Week 5	401.7 ± 21.7	395.1 ± 32.8	413.4 ± 34.8	393.0 ± 18.7
	Week 6	435.9 ± 26.2	439.4 ± 41.9	459.0 ± 37.2	434.3 ± 25.1
	Week 7	478.9 ± 25.6	472.4 ± 47.2	500.5 ± 44.9	465.1 ± 22.4
	Week 8	498.4 ± 28.3	496.0 ± 49.8	520.5 ± 44.3	493 ± 29.5

Week 9	518.7 ± 24.7	524.2 ± 59.1	542.8 ± 43.3	513.1 ± 35.9
Week 10	539.8 ± 28.6	540.9 ± 56.5	557.3 ± 46.6	532.5 ± 42.1
Week 11	552.9 ± 28.8	554.3 ± 60.7	573.1 ± 44.8	548.4 ± 47.0
Week 12	569.2 ± 32.2	571.1 ± 62.7	588.0 ± 47.7	566.3 ± 46.6
End of the treatment period	576.7 ± 33.8	582.0 ± 67.0	595.2 ± 51.0	575.6 ± 46.6
Total weight gain	496.9 ± 34.5	501.9 ± 66.2	515.2 ± 51.1	495.0 ± 48.3

Note: *indicates a statistically significant difference compared with the control group (P < 0.05)

Table 5 Statistical Summary Table of Animal Food Consumption in Each Dose Group of the 90-Day Oral Toxicity Study in Rats (g, $\overline{x}$ ± s)

Sex	Time	Dose Groups (n=10)			
		Control Group	Low Dose Group	Intermediate-dose Group	High Dose Group
Female	Week 1	139.5 ± 6.6	140.6 ± 12.4	131.3 ± 16.0	150.7 ± 7.6*
	Week 2	174 ± 18.4	166.2 ± 21.0	159.5 ± 22.1	170.2 ± 9.8
	Week 3	193.9 ± 30.9	182.5 ± 29.1	174.6 ± 25.9	175.2 ± 15.7
	Week 4	182.1 ± 14.6	195.5 ± 47.7	178.6 ± 24.0	184.8 ± 20.6
	Week 5	176.7 ± 15.3	177.9 ± 29.6	174.4 ± 17.9	177.6 ± 22.8
	Week 6	187 ± 17.5	191.7 ± 35.2	172.8 ± 23.2	187.1 ± 23.8
	Week 7	181.6 ± 21.4	178.1 ± 32.2	165.7 ± 19.9	176.8 ± 23.0
	Week 8	181.8 ± 19.4	182.5 ± 34.0	171.2 ± 28.4	183.9 ± 21.3
	Week 9	174.8 ± 20.6	172.9 ± 23.3	166.2 ± 26.0	175.7 ± 23.7
	Week 10	184.2 ± 12.2	177.4 ± 25.2	161.1 ± 22.4	174.8 ± 28.0
	Week 11	184.1 ± 16.2	169.8 ± 20.33	164 ± 29.9	167.2 ± 26.4
	Week 12	177 ± 20.1	159.6 ± 16.0	157 ± 25.5	175.4 ± 27.2
	Week 13	129.2 ± 16.1	118.7 ± 12.4	118.2 ± 20.8	120.4 ± 16.4
	Total food intake	2265.8 ± 207.3	2213.5 ± 327.7	2094.5 ± 285.5	2219.6 ± 245.1
Male	Week 1	159.2 ± 7.3	140 ± 15.3**	145.5 ± 10.4*	145.7 ± 11.2*
	Week 2	199.2 ± 17.1	179.2 ± 21.3*	179.3 ± 17.8*	180.8 ± 13.3
	Week 3	243.4 ± 13.3	221.1 ± 21.6	235.8 ± 19.7	232 ± 18.9
	Week 4	254.7 ± 20.4	240.7 ± 24.3	253.6 ± 26.1	252.8 ± 19.9
	Week 5	259.7 ± 24.5	246 ± 23.9	249.9 ± 34.1	241.5 ± 24.7
	Week 6	270.5 ± 19.7	248.3 ± 29.7	264.4 ± 27.5	257.8 ± 25.0

	Week 7	242.2 ± 37.2	239.4 ± 29.4	254.8 ± 32.4	226.3 ± 40.7
	Week 8	254.7 ± 30.2	233.3 ± 30.6	236.3 ± 26.9	240.9 ± 21.4
	Week 9	238 ± 28.9	239.6 ± 28.5	233.7 ± 21.8	230.8 ± 24.6
	Week 10	256.6 ± 35.7	222.4 ± 27.1	226.8 ± 21.5	227.7 ± 28.4
	Week 11	232.7 ± 28.3	224.5 ± 22.9	230.9 ± 20.6	229.7 ± 23.3
	Week 12	228.3 ± 26.7	223.6 ± 30.3	228.4 ± 23.0	224.1 ± 21.1
	Week 13	167.3 ± 19.2	165.5 ± 25.4	170.5 ± 17.6	159.1 ± 17.6
	Total food intake	3006.5 ± 264.8	2823.5 ± 310.7	2909.8 ± 277.9	2849.1 ± 225.5

Note: *indicates a statistically significant difference compared with the control group ($P < 0.05$); ** indicates a statistically significant difference compared with the control group ($P < 0.01$).

Table 6 Statistical Summary Table of Food Utilization Rate in Each Dose Group of the 90-Day Oral Toxicity Study in Rats (%, $\overline{x} \pm s$)

Sex	Time	Dose Groups (n=10)			
		Control Group	Low Dose Group	Intermediate-dose Group	High Dose Group
Female	Week 1	39.1 ± 6.0	44.2 ± 3.2	42.0 ± 5.6	39.1 ± 4.1
	Week 2	27.1 ± 5.2	27.8 ± 4.1	30.5 ± 3.0	26.7 ± 5.0
	Week 3	16.1 ± 2.6	18.6 ± 4.8	18.1 ± 2.6	14.6 ± 3.9
	Week 4	13.0 ± 3.3	14.6 ± 4.2	15.7 ± 2.9	12.7 ± 3.3
	Week 5	11.1 ± 6.1	11.4 ± 4.6	9.2 ± 2.8	10.7 ± 5.0
	Week 6	7.0 ± 4.5	8.7 ± 5.3	9.7 ± 4.1	9.0 ± 3.7
	Week 7	5.2 ± 2.3	8.1 ± 5.0	9.7 ± 4.5*	4.2 ± 3.9
	Week 8	4.3 ± 2.7	5.1 ± 3.4	3.9 ± 2.3	6.0 ± 3.5
	Week 9	8.0 ± 4.6	8.0 ± 4.5	7.2 ± 3.3	7.3 ± 3.3
	Week 10	3.8 ± 3.2	2.6 ± 2.9	2.3 ± 2.3	3.8 ± 1.6
	Week 11	5.2 ± 4.5	2.8 ± 2.2	3.1 ± 3.6	4.0 ± 4.0
	Week 12	1.9 ± 3.2	2.4 ± 3.6	2.5 ± 1.8	5.1 ± 3.4
	Week 13	0.4 ± 2.9	1.7 ± 4.4	2.8 ± 4.5	1.6 ± 4.3
	Total food utilization rate	10.6 ± 1.4	11.7 ± 1.1	11.8 ± 1.0	10.8 ± 1.1
Male	Week 1	43.7 ± 4.0	47.3 ± 3.7	46.3 ± 3.7	43.3 ± 4.3
	Week 2	34.4 ± 4.9	38.3 ± 1.8	39.3 ± 2.3*	38.4 ± 4.8
	Week 3	28.4 ± 1.8	30.1 ± 2.9	32.5 ± 3.0**	28.6 ± 2.1

Week 4	24.1 ± 3.7	26.2 ± 2.2	24.4 ± 3.7	25.2 ± 3.3
Week 5	21.0 ± 1.5	20.8 ± 4.3	22.9 ± 4.0	20.9 ± 3.5
Week 6	12.6 ± 6.1	17.7 ± 3.2	17.4 ± 1.8	16.1 ± 3.2
Week 7	16.5 ± 2.8	13.7 ± 3.5	16.1 ± 1.9	13.8 ± 3.6
Week 8	7.5 ± 2.7	10.1 ± 3.1	8.7 ± 5.0	11.5 ± 4.5
Week 9	8.7 ± 3.2	11.5 ± 3.0	9.6 ± 4.3	8.6 ± 3.4
Week 10	8.4 ± 3.6	7.7 ± 5.5	6.4 ± 2.1	8.4 ± 3.6
Week 11	5.7 ± 2.8	5.9 ± 4.3	6.9 ± 4.1	6.7 ± 3.8
Week 12	7.2 ± 3.2	7.5 ± 4.5	6.5 ± 4.5	8.1 ± 2.2
Week 13	4.4 ± 1.7	6.4 ± 3.8	4.2 ± 3.8	6.0 ± 2.6
Total food utilization rate	16.5 ± 1.0	17.8 ± 1.0**	17.7 ± 0.6*	17.4 ± 0.9

Note: * indicates a statistically significant difference compared with the control group ($P < 0.05$); ** indicates a statistically significant difference compared with the control group ($P < 0.01$).

Table 7 Summary of Recovery High-dose Satellite Group Body Weights of Rats in the 90-day Oral Toxicity Test (g, $\overline{x} \pm s$)

Sex	Time (week)	Dose group (n=5)	
		Recovery control satellite group	Recovery high-dose satellite group
Female	Initial body weight	76.1 ± 3.3	77.9 ± 7.3
	1	134.0 ± 3.6	138.6 ± 12.8
	2	176.3 ± 4.2	186.7 ± 12.0
	3	204.9 ± 8.1	213.8 ± 17.7
	4	226.9 ± 7.8	240.6 ± 15.8
	5	242.6 ± 10.7	254.1 ± 13.4
	6	256.3 ± 12.5	276.8 ± 25.8
	7	270.2 ± 12.6	294.1 ± 25.7
	8	276.2 ± 13.1	302.9 ± 30.3
	9	283.6 ± 11.7	317.5 ± 35.3
	10	291.9 ± 14.6	319.5 ± 33.7
	11	303.2 ± 17.1	324.9 ± 31.5
	12	306.2 ± 18.2	330.0 ± 33.1
	13	309.0 ± 20.3	334.5 ± 37.6
	14	318.4 ± 22.2	341.1 ± 38.5

	15	328.1 ± 20.5	344.9 ± 41.5
	16	333.3 ± 23.8	349.1 ± 42.6
	17	338.5 ± 27.0	353.6 ± 45.6
	Total weight gain	262.4 ± 26.6	275.7 ± 39.3
Male	Initial body weight	79.3 ± 5.2	79.9 ± 3.9
	1	142.8 ± 10.6	143.7 ± 5.0
	2	211.6 ± 16.5	223.0 ± 13.1
	3	275.6 ± 20.0	297.2 ± 21.7
	4	339.0 ± 24.4	366.7 ± 35.3
	5	388.2 ± 26.2	422.7 ± 36.1
	6	427.2 ± 36.2	465.4 ± 40.7
	7	460.4 ± 42.3	496.2 ± 42.2
	8	482.9 ± 46.9	525.9 ± 39.3
	9	502.8 ± 48.1	550.0 ± 47.2
	10	521.2 ± 50.3	568.1 ± 47.2
	11	540.4 ± 58.2	584.1 ± 49.4
	12	553.9 ± 57.7	597.9 ± 49.3
	13	560.7 ± 59.0	609.0 ± 48.4
	14	575.3 ± 55.1	621.3 ± 52.4
	15	588.7 ± 62.1	630.6 ± 53.9
	16	596.6 ± 62.8	644.4 ± 57.1
	17	600.2 ± 61.6	651.0 ± 56.7
	Total weight gain	521.0 ± 61.2	571.0 ± 56.3

Table 8 Summary of Recovery High-dose Satellite Group Food Consumption of Rats in the 90-day Oral Toxicity Test (g, $\overline{x}$ ± s)

Sex	Time (week)	Dose group (n=5)	
		Recovery control satellite group	Recovery high-dose satellite group
Female	1	132.1 ± 3.8	140.1 ± 9.8
	2	157.8 ± 3.2	166.5 ± 12.5
	3	162.3 ± 4.1	169.5 ± 8.1
	4	167.0 ± 7.1	176.9 ± 9.9

	5	156.8 ± 5.2	173.2 ± 7.9**
	6	180.2 ± 23.2	181.3 ± 10.7
	7	162.8 ± 8.5	188.9 ± 10.4**
	8	163.7 ± 7.7	167.6 ± 8.4
	9	162.4 ± 7.5	172.0 ± 11.3
	10	157.3 ± 5.3	168.3 ± 14.3
	11	167.6 ± 6.0	192.1 ± 32.3
	12	153.2 ± 6.3	159.5 ± 12.6
	13	117.9 ± 4.8	117.6 ± 6.4
	14	196.0 ± 10.5	195.7 ± 16.0
	15	210.2 ± 10.5	195.1 ± 17.8
	16	149.1 ± 16.7	147.0 ± 19.3
	17	120.1 ± 9.4	123.6 ± 10.3
	Total food consumption (g)	2506.3 ± 81.6	2639.8 ± 140.8
Male	1	148.8 ± 5.8	152.9 ± 4.1
	2	189.9 ± 14.4	194.0 ± 10.6
	3	230.4 ± 22.0	257.3 ± 12.2*
	4	247.5 ± 23.3	266.6 ± 16.8
	5	245.5 ± 23.6	261.7 ± 19.8
	6	255.0 ± 21.9	269.2 ± 20.2
	7	246.1 ± 21.0	273.5 ± 30.7
	8	232.7 ± 25.9	262.2 ± 34.2
	9	218.1 ± 24.0	253.6 ± 22.9*
	10	222.6 ± 28.6	253.5 ± 23.3
	11	224.9 ± 30.5	245.3 ± 25.4
	12	224.1 ± 25.0	229.9 ± 29.6
	13	156.3 ± 16.6	174.2 ± 21.0
	14	263.2 ± 34.9	287.5 ± 31.6
	15	251.7 ± 50.3	268.6 ± 76.3
	16	214.0 ± 27.5	253.8 ± 22.0*

17	159.1 ± 19.5	178.1 ± 15.5
Total food consumption (g)	3478.2 ± 353.7	3813.4 ± 304.0

Note: * indicates that the difference was statistically significant compared with the control group (P<0.05); ** indicates that the difference was statistically significant compared with the control group (P<0.01).

Table 9 Summary of Recovery High-dose Satellite Group Food Utilization of Rats in the 90-day Oral Toxicity Test (g, $\overline{x}$ ± s)

Sex	Time (week)	Dose group (n=5)	
		Recovery control satellite group	Recovery high-dose satellite group
Female	1	43.8 ± 1.7	43.3 ± 1.9
	2	26.9 ± 4.4	29.1 ± 2.5
	3	17.6 ± 4.0	15.9 ± 4.2
	4	13.3 ± 5.7	15.3 ± 4.4
	5	10.0 ± 5.9	7.9 ± 3.0
	6	7.9 ± 3.4	12.2 ± 6.5
	7	8.5 ± 2.6	9.2 ± 0.9
	8	3.7 ± 3.4	5.2 ± 2.7
	9	4.6 ± 2.3	8.4 ± 2.9
	10	5.2 ± 2.8	1.2 ± 1.5*
	11	6.7 ± 1.9	3.2 ± 3.7
	12	2.0 ± 1.2	2.6 ± 1.1
	13	2.3 ± 2.3	3.6 ± 4.7
	14	4.8 ± 3.0	3.3 ± 2.6
	15	4.6 ± 3.0	1.8 ± 1.4
	16	3.5 ± 2.6	2.8 ± 1.3
	17	4.1 ± 2.8	3.6 ± 3.0
	Total food utilization	10.5 ± 0.8	10.4 ± 1.1
Male	1	42.7 ± 4.7	41.7 ± 2.9
	2	36.1 ± 2.1	41.0 ± 5.3
	3	27.9 ± 2.7	28.8 ± 2.5
	4	25.6 ± 1.0	25.9 ± 4.9

5	20.2 ± 1.7	21.5 ± 2.0
6	15.1 ± 3.4	15.8 ± 1.9
7	13.4 ± 1.9	11.2 ± 2.3
8	9.6 ± 1.2	11.4 ± 1.5
9	9.2 ± 2.2	9.4 ± 3.3
10	8.2 ± 1.9	7.2 ± 2.0
11	8.3 ± 5.2	6.4 ± 3.0
12	6.1 ± 3.7	5.5 ± 1.4
13	4.3 ± 2.2	6.5 ± 1.6
14	5.9 ± 3.3	5.4 ± 2.1
15	5.1 ± 2.6	4.1 ± 3.1
16	3.6 ± 1.9	5.4 ± 4.4
17	2.4 ± 2.2	3.7 ± 2.3
Total food utilization	15.0 ± 0.7	15.0 ± 1.0

Note: * indicates that the difference was statistically significant compared with the control group ($P<0.05$).
