# Supplementary material for: Evaluation of the safety and functional effects of recombinant humanized type III collagen in food toxicology
Source: Front Med (Lausanne). 2026 Feb 12;13:1765276. doi: 10.3389/fmed.2026.1765276 (PMC12936025; doi:10.3389/fmed.2026.1765276)
Supplement: Supplementary file 2 [file Data_Sheet_2.pdf]

Appendix B

Gross Anatomy and Histopathological Data of Rats

Table 1 Histological Effects of Recombinant Type III Collagen on Rat Brain

Lesions (Cases)	Control Group (n=20)	High-dose Group (n=20)
Capsule Changes	0	0
Neuronal Shrinkage, Triangular Nucleus, Condensed and Hyperchromatic Nissl Bodies	0	0
Neuronal Swelling	0	0
Neuronal Liquefactive Necrosis	0	0
Neuronal Lipofuscin Deposition	0	0
Degenerative and Progressive Changes in Neuroglial Cells	0	0

Note: n represents the number of animals per group.

Table 2 Histological Effects of Recombinant Type III Collagen on Rat Pituitary

Lesions (Cases)	Control Group (n=20)	High-dose Group (n=20)
Interstitial inflammatory cell infiltration	0	0
Cyst	0	0
Vascular dilatation	0	0
Hypertrophy	0	0
Hyperplasia	0	0
Necrosis	0	0

Note: n represents the number of animals per group.

Table 3 Histological Effects of Recombinant Type III Collagen on Rat Thyroid

Lesions (Cases)	Control Group (n=20)	High-dose Group (n=20)
Reduced function, enlarged follicles containing abundant and dense colloid	0	0
Increased function, small follicles with lightly stained colloid	0	0
Follicular cell hyperplasia	0	0
Follicular cell atrophy	0	0
Follicular cystic degeneration	0	0
Residual ultimobranchial body	0	0

Note: n represents the number of animals per group.

Table 4 Histological Effects of Recombinant Type III Collagen on Rat Thymus

Lesions (Cases)	Control Group (n=20)	High-dose Group (n=20)
Capsule changes	0	0
Intrathymic tubular structures	0	0
Thymic atrophy	0	0
Thymic congestion	0	0
Cortical thinning, decreased lymphocytes, and macrophage hyperplasia	0	0

Note: n represents the number of animals per group.

Table 5 Histological Effects of Recombinant Type III Collagen on Rat Lung

Lesions (Cases)	Control Group (n=20)	High-dose Group (n=20)
Accumulation of foamy alveolar macrophages	0	0
Slight hyperplasia of type II alveolar epithelial cells	0	0
Degeneration/necrosis of bronchial mucosal epithelial cells	0	0
Vascular dilatation and congestion in alveolar walls	0	0
Interstitial edema and inflammatory cell infiltration	0	0
Loss of lung architecture with consolidation; neutrophils in lumen	0	0
Proliferation of capillaries and fibroblasts in alveolar spaces	0	0
Thickened alveolar walls with proliferation/aggregation of mononuclear macrophages	0	0
Focal pulmonary hemorrhage	0	0
Alveolar dilation with narrowed septa	0	0

Note: n represents the number of animals per group.

Table 6 Histological Effects of Recombinant Type III Collagen on Rat Heart

Lesions (Cases)	Control Group (n=20)	High-dose Group (n=20)
Myocardial hypertrophy	0	0
Cardiac myocyte degeneration and necrosis	0	0
Endocardial hyperplasia	0	0
Diffuse congestion	0	0
Nodular periarteritis	0	0

Note: n represents the number of animals per group

Table 7 Histological Effects of Recombinant Type III Collagen on Rat Liver

Lesions (Cases)	Control Group (n=20)	High-dose Group (n=20)
Capsule changes	0	0

Hepatic congestion and edema	0	0
Scattered punctate necrosis in hepatic lobules	0	0
Scattered focal necrosis in hepatic lobules	0	0
Hydropic degeneration and eosinophilic change in hepatic lobules	0	0
Fibrous tissue hyperplasia and small bile duct hyperplasia	0	0
Mild fatty degeneration in hepatic lobules	0	0
Inflammatory cell infiltration in portal areas	0	0
Extramedullary hematopoiesis	0	0

Table 8 Histological Effects of Recombinant Type III Collagen on Rat Spleen

Lesions (Cases)	Control Group (n=20)	High-dose Group (n=20)
Red pulp dilation, fibrous tissue hyperplasia	0	0
Atrophy of splenic corpuscles	0	0
Pigmentation	0	0
Splenic sinusoid dilation, congestion, and inflammatory cell infiltration	0	0
Macrophage hyperplasia	0	0
Granuloma formation	0	0
Lymphoid tissue hyperplasia	0	0
Extramedullary hematopoiesis	0	0

Table 9 Histological Effects of Recombinant Type III Collagen on Rat Kidney

Lesions (Cases)	Control Group (n=20)	High-dose Group (n=20)
Capsule changes	0	0
Glomerular cell hyperplasia, degeneration, and inflammatory cell infiltration	0	0
Renal tubular degeneration, necrosis, and casts within renal tubules	0	0
Glomerular hyaline degeneration and sclerosis	0	0
Focal inflammatory cell infiltration and fibrous hyperplasia in the renal interstitium	0	0
Focal dilation of renal tubules and collecting ducts	0	0
Focal degeneration of renal tubular cells and collecting duct cells	0	0
Renal mucosal degeneration, inflammatory cell infiltration in submucosal tissue, and fibrous hyperplasia	0	0
Simple cysts	0	0
Mild hyperplasia of renal pelvic epithelial cells	0	0

Table 10 Histological Effects of Recombinant Type III Collagen on Rat Adrenal Gland

Lesions (Cases)	Control Group (n=20)	High-dose Group (n=20)

Capsule changes	0	0
Cortical atrophy, with the zona glomerulosa and zona reticularis atrophied and replaced by adipocytes	0	0
Medullary atrophy, decreased cells, and vacuolation	0	0
Adrenal congestion	0	0
Ossification of the adrenal cortex	0	0
Capsule changes	0	0

Table 11 Histological Effects of Recombinant Type III Collagen on Rat Stomach

Lesions (Cases)	Control Group (n=20)	High-dose Group (n=20)
Degeneration and necrosis of the mucosal epithelium	0	0
Intestinal metaplasia of the mucosal epithelium	0	0
Inflammatory cell infiltration in the lamina propria	0	0
Reduced glands in the lamina propria	0	0
Glandular hypertrophy and hyperplasia in the lamina propria	0	0

Table 12 Histological Effects of Recombinant Type III Collagen on Rat Duodenum

Lesions (Cases)	Control Group (n=20)	High-dose Group (n=20)
Degeneration and necrosis of the mucosal epithelium	0	0
Congestion and edema in the submucosa	0	0
Inflammatory cell infiltration in all layers of the intestinal wall	0	0
Granuloma formation	0	0

Table 13 Histological Effects of Recombinant Type III Collagen on Rat Large Intestine and Colon

Lesions (Cases)	Control Group (n=20)	High-dose Group (n=20)
Degeneration and necrosis of mucosal epithelial cells	0	0
Edema, purple spots, and hemorrhage of the mucosa	0	0
Inflammatory cell infiltration in all layers of the intestinal wall	0	0
Granuloma formation	0	0

Table 14 Histological Effects of Recombinant Type III Collagen on Rat Pancreas

Lesions (Cases)	Control Group (n=20)	High-dose Group (n=20)
Pancreatic islet cell hyperplasia	0	0
Acinar cell atrophy	0	0
Ductal hyperplasia	0	0
Vacuolar degeneration of acinar cells	0	0
Pancreatic islet cell adenoma	0	0

Table 15 Histological Effects of Recombinant Type III Collagen on Rat Mesenteric Lymph Nodes

Lesions (Cases)	Control Group (n=20)	High-dose Group (n=20)
Congestion	0	0

Histiocyte hyperplasia	0	0
Cortical atrophy with disappearance of lymphoid follicles	0	0
Focal vesicular dilation of lymphatic sinuses	0	0

Table 16 Histological Effects of Recombinant Type III Collagen on Rat Testis

Lesions (Cases)	Control Group (n=10)	High-dose Group (n=10)
Decrease in spermatogenic cells	0	0
Degeneration and necrosis of spermatogenic cells	0	0
Multinucleated giant cells	0	0
Inflammatory cell infiltration in the interstitial tissue	0	0
Interstitial edema	0	0

Table 17 Histological Effects of Recombinant Type III Collagen on Rat Epididymis

Lesions (Cases)	Control Group (n=10)	High-dose Group (n=10)
Decrease in spermatogenic cells	0	0
Degeneration and necrosis of spermatogenic cells	0	0
Multinucleated giant cells	0	0
Inflammatory cell infiltration in the interstitial tissue	0	0
Interstitial edema	0	0

Table 18 Histological Effects of Recombinant Type III Collagen on Rat Ovary

Lesions (Cases)	Control Group (n=10)	High-dose Group (n=10)
Ovarian atrophy	0	0
Ovarian abscess	0	0
Ovarian cyst	0	0
Hyperplasia of stromal glands and stromal cells	0	0

Table 19 Histological Effects of Recombinant Type III Collagen on Rat Uterus

Lesions (Cases)	Control Group (n=10)	High-dose Group (n=10)
Squamous metaplasia	0	0
Endometrial hyperplasia	0	0
Endometritis	0	0
Uterine tumor	0	0

Table 20 Histological Effects of Recombinant Type III Collagen on Rat Bladder

Lesions (Cases)	Control Group (n=20)	High-dose Group (n=20)
Degeneration and necrosis of mucosal epithelial cells	0	0
Lymphocytic infiltration in the mucosal lamina propria	0	0
Cystitis	0	0
Mild sediment in the bladder	0	0
Bladder hemorrhage	0	0

Table 21 Histological Effects of Recombinant Type III Collagen on Rat Prostate

Lesions (Cases)	Control Group (n=10)	High-dose Group (n=10)
Hyperplasia of prostate epithelial cells	0	0
Inflammatory cell infiltration in the prostate	0	0
Prostatic atrophy and degeneration	0	0
Prostatic adenoma	0	0
Adenocarcinoma of the prostate	0	0
