# Supplementary material for: Evaluation of the safety and functional effects of recombinant humanized type III collagen in food toxicology
Source: Front Med (Lausanne). 2026 Feb 12;13:1765276. doi: 10.3389/fmed.2026.1765276 (PMC12936025; doi:10.3389/fmed.2026.1765276)
Supplement: Supplementary file 3 [file Data_Sheet_3.pdf]

Appendix C

Zebrafish Preliminary Experiment

1. Anti-aging assay

1.1 Determination of the Maximum Tolerated Concentration (MTC)

The sample stock solution was serially diluted in a 2-fold geometric series using the modeling agent (20 mg/mL D-galactose) as the diluent (concentrations are listed in Table 1-1). Normally developing zebrafish embryos at 6 hours post-fertilization (6 hpf) were selected and transferred into 6-well plates containing the corresponding diluted solutions (30 embryos per well). Without causing harm to the embryos/larvae, the residual solution in the wells was removed, the plate was covered and wrapped with aluminum foil, and then incubated in a biochemical incubator at (28.5 ± 1.0) °C under dark conditions. After 5 days of incubation, larvae were observed; during the incubation period, fresh test solutions were replaced daily. The MTC of the sample in zebrafish was then determined.

1.2 Maximum Tolerated Concentration (MTC)

Under the experimental conditions, the MTC of the sample in the zebrafish anti-aging efficacy assay was 250 μg/mL. The results are shown in Table 1-1.

Table 1 Pilot Concentration-finding Results of Zebrafish Anti-aging Efficacy Assay (n = 30)

Concentration (μg/mL)	No. of deaths (fish)	Mortality (%)	Phenotype
62.5	0	0	Similar to the normal control group
125	0	0	Similar to the normal control group
250	0	0	Similar to the normal control group
500	0	0	Worse than the normal control group
1000	0	0	Worse than the normal control group

2. Anti-glycation assay

2.1 Determination of the Maximum Tolerated Concentration (MTC)

The sample stock solution was serially diluted in a 2-fold geometric series using the modeling agent (0.4 M glucose) as the diluent (concentrations are listed in Table 2-1). Normally developing zebrafish larvae at 5 days post-fertilization (5 dpf) were selected and transferred into 6-well plates containing the corresponding diluted solutions (30 larvae per well). Without causing harm to the larvae, the residual solution in the wells was removed, the plate was covered and wrapped with aluminum foil, and then incubated in a biochemical incubator at (28.5 ± 1.0) °C under dark conditions. After 3 days of incubation, larvae were observed; during the incubation period, fresh test

solutions were replaced daily. The MTC of the sample in zebrafish was then determined.

2.2 Maximum Tolerated Concentration (MTC)

Under the experimental conditions, the MTC of the sample in the zebrafish anti-glycation efficacy assay was 250 μg/mL. The results are shown in Table 2-1.

Table 2 Pilot Concentration-finding Results of the Zebrafish Anti-glycation Efficacy assay (n = 30)

Concentration (μg/mL)	No. of deaths (fish)	Mortality (%)	Phenotype
62.5	0	0	Similar to the normal control group
125	0	0	Similar to the normal control group
250	0	0	Similar to the normal control group
500	0	0	Worse than the normal control group
1000	0	0	Worse than the normal control group

3. Antioxidant assay

3.1 Determination of the Maximum Tolerated Concentration (MTC)

The sample stock solution was serially diluted in a 2-fold geometric series using the modeling agent (600 μM hydrogen peroxide, H_2O_2) as the diluent (concentrations are listed in Table 3-1). Normally developing zebrafish larvae at 3 days post-fertilization (3 dpf) were selected and transferred into 6-well plates containing the corresponding diluted solutions (30 larvae per well). Without causing harm to the larvae, the residual solution in the wells was removed, the plate was covered and wrapped with aluminum foil, and then incubated in a biochemical incubator at (28.5 ± 1.0) °C under dark conditions. After 24 h of incubation, larvae were observed, and the MTC of the sample in zebrafish was determined.

3.1 Maximum Tolerated Concentration (MTC)

Under the experimental conditions, the MTC of the sample in the zebrafish antioxidant efficacy assay was 250 μg/mL. The results are shown in Table 3-1.

Table 3 Pilot Concentration-finding Results of the Zebrafish Antioxidant Efficacy Assay (n = 30)

Concentration (μg/mL)	No. of deaths (fish)	Mortality (%)	Phenotype
62.5	0	0	Similar to the normal control group
125	0	0	Similar to the normal control group
250	0	0	Similar to the normal control group
500	0	0	Worse than the normal control group
1000	0	0	Worse than the normal control group

4. Anti-inflammatory assay

4.1 Determination of the Maximum Tolerated Concentration (MTC)

The sample stock solution was serially diluted in a 2-fold geometric series using the modeling agent (60 μg/mL sodium dodecyl sulfate, SDS) as the diluent (concentrations are listed in Table 4-1). Normally developing zebrafish larvae at 2 days post-fertilization (2 dpf) were selected and transferred into 6-well plates containing the corresponding diluted solutions (30 larvae per well). Without causing harm to the larvae, the residual solution in the wells was removed, the plate was covered and wrapped with aluminum foil, and then incubated in a biochemical incubator at (28.5 ± 1.0) °C under dark conditions. After 24 h of incubation, larvae were observed, and the MTC of the sample in zebrafish was determined.

4.2 Maximum Tolerated Concentration (MTC)

Under the experimental conditions, the MTC of the sample in the zebrafish sensitive-skin (anti-inflammatory) efficacy assay was 500 μg/mL. The results are shown in Table 4-1.

Table 4 Pilot Concentration-finding Results of the Zebrafish Sensitive-skin (anti-inflammatory) Efficacy Assay (n = 30)

Concentration (μg/mL)	No. of deaths (fish)	Mortality (%)	Phenotype
62.5	0	0	Similar to the normal control group
125	0	0	Similar to the normal control group
250	0	0	Similar to the normal control group
500	0	0	Similar to the normal control group
1000	0	0	Worse than the normal control group
