# Supplementary material for: Evaluation of the safety and functional effects of recombinant humanized type III collagen in food toxicology
Source: Front Med (Lausanne). 2026 Feb 12;13:1765276. doi: 10.3389/fmed.2026.1765276 (PMC12936025; doi:10.3389/fmed.2026.1765276)
Supplement: Supplementary file 4 [file Data_Sheet_4.pdf]

Appendix D

Zebrafish Anti-Aging, Anti-Glycation, Antioxidant, and Anti-Inflammatory Efficacy Test Data

Table 1 Anti-Aging Action, β-galactosidase Activity % (n=10)

Group	Normal control group	Model group	Positive control group	Sample set		
				5 μg/mL	50 μg/mL	500 μg/mL
Average	360227.50	583035.60	424759.90	507152.20	481017.30	457270.90
SD-value	29789.98	78432.06	52856.71	16935.88	41880.89	69078.73
P-value	/	< 0.0001	0.0001	0.0179	0.0041	0.0020

Table 2 Anti-Glycation Action, AGEs Fluorescence Intensity (n=10)

Group	Normal control group	Model group	Positive control group	Sample set		
				5 μg/mL	50 μg/mL	250 μg/mL
Average	3030.90	3821.20	3104.90	3513.00	3155.00	3145.90
SD-value	138.26	298.96	273.19	281.22	387.00	300.06
P-value	/	<0.0001	<0.0001	0.037	0.0008	0.0001

Table 3 Anti-Oxidation Action, ROS Fluorescence Intensity (n=10)

Group	Normal control group	Model group	Positive control group	Sample set		
				5 μg/mL	50 μg/mL	250 μg/mL
Average	1500.70	2857.10	1667.50	2484.70	2373.30	1993.80
SD-value	239.46	438.45	279.44	318.28	240.21	77.29
P-value	/	<0.0001	<0.0001	0.0554	0.0116	0.0002

Table 4 Anti-Inflammatory Action, Statistics of Neutrophils in Each Group (n=10)

Group	Normal control group	Model group	Positive control group	Sample set		
				10 μg/mL	100 μg/mL	500 μg/mL
Average	3.90	35.90	4.30	27.80	21.00	21.30
SD-value	1.87	9.15	2.76	5.53	4.67	5.44
P-value	/	<0.0001	<0.0001	0.0383	0.0007	0.0010
